# Research advances and public health strategies in China on WHO priority fungal pathogens

**DOI:** 10.1080/21501203.2025.2561612

**Published:** 2025-09-29

**Authors:** Yue Wang, Li Han, Jie Gong, Liu Liu, Beibei Miao, Jianping Xu

**Affiliations:** a Department of Biology, McMaster University, Hamilton, Canada; b Institute of Microbiology, Chinese Academy of Sciences, Beijing, China; c National Key Laboratory of Intelligent Tracking and Forecasting for Infectious Diseases, National Institute for Communicable Disease Control and Prevention, Chinese Center for Disease Control and Prevention, Beijing, China; d National Institute for Communicable Disease Control and Prevention Joint Laboratory of Pathogenic Fungi, Peking University First Hospital, Beijing, China

**Keywords:** WHO, China, fungal pathogens, research trends, public health

## Abstract

Fungal pathogens pose significant and increasing threats to public health. Each year, over a billion people are infected by fungal pathogens, directly contributing to millions of deaths. To overcome the challenge of fungal threat, in 2022, World Health Organization (WHO) issued a Fungal Priority Pathogens List (FPPL) aimed at strengthening international response, promoting research, and enhancing policy intervention development. Over the past four decades, China has made tremendous progress in advancing our knowledge of fungal infections. Here, we review research trends and recent progress in China on fungal pathogens on the WHO FPPL, with an emphasis on four critical pathogens: *Cryptococcus neoformans*, *Candidozyma auris*, *Aspergillus fumigatus*, and *Candida albicans* since 2022. In addition, we describe national policies and strategic measures aimed at large-scale prevention and control of fungal infections. Our bibliometric analyses of articles published by Chinese researchers from 1983 to 2024 in the Web of Science Core Collection (WOSCC, English-language) and China National Knowledge Infrastructure (CNKI, Chinese-language) revealed increasing number of peer-reviewed publications on human fungal pathogens in both databases up to 2008 when the number in the CKNI database dropped and remained relatively flat since while that in the WOSCC database continued to increase, reflecting the strategic emphasis by Chinese institutions and funding agencies on achieving greater international visibility, academic impact, and integration within the global scientific community. In both databases, the four critical priority pathogens accounted for > 45% of the studies and the progresses made by Chinese researchers since 2022 on them are described here. A shared challenge for treating all fungal infections is the emergence and spread of antifungal resistance. We highlight antifungal resistance, tolerance, and persistence, and describe recent developments in antifungal drug pipelines, including those in China. Beyond scientific breakthroughs, China has been making coordinated prevention efforts and robust policy measures. However, significant challenges remain in understanding pathogen population dynamics and host-pathogen interactions; in developing and deploying rapid, sensitive, specific, and cost-effective diagnosis; in designing geographic region-specific and personalized prevention and treatments; and in alleviating the growing burden of antifungal resistance.

## GRAPHICAL ABSTRACT

**Figure:**
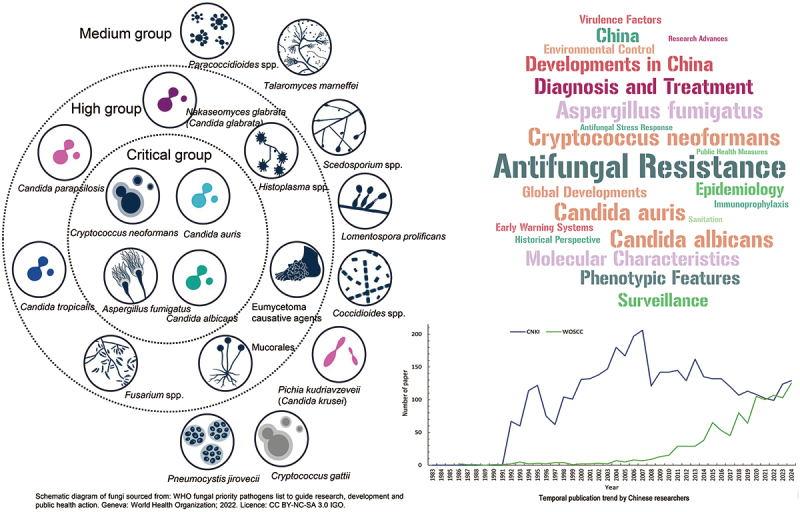

## 1. Introduction

Fungal pathogens cause a wide range of infections ranging from mild skin and mucosal infections to severe invasive, systemic, and disseminated forms (Xu 2022). Compared to bacterial and viral infections, fungal infections are typically more difficult to diagnose due to the slow growth of many fungal pathogens and the lack of specificity in common diagnostic methods. In addition, as eukaryotes, fungi share many features with human cells, complicating drug treatments. The situation is further complicated by limited drug options and the growing emergence of antifungal resistance.

Fungal infections are prone to chronicity, recurrence, and persistence, particularly in immunocompromised individuals (Pathakumari et al. 2020). Rayens et al. (2022) analyzed data from the National Vital Statistics System (1999–2018) in the United States and reported several trends related to fungal disease and the associated risk factors. Overall, there has been a decreasing trend in fungal disease among patients with acquired immunodeficiency syndrome (AIDS), largely attributed to the utilization of antiretroviral therapies for AIDS control. However, there is an overall increase in fungal disease incidences, and those with cancer, sepsis, immunosuppressive disorders, and influenza are especially at high risk of developing serious fungal infections. Moreover, the number of deaths due to fungal disease in non-AIDS group is rising, particularly in patients with diabetes, cancers, immunosuppressive disorders, or sepsis (Rayens et al. 2022).

Severe fungal diseases pose an escalating threat to global public health. Denning (2024) estimated that annually, more than 6.5 million people suffer from invasive fungal and chronic pulmonary aspergillosis, with an overall crude mortality reaching 3.8 million. Among the major fungal pathogens, *Aspergillus* spp. was estimated to cause 2,113,000 (mortality rate: 85.2%) cases of invasive aspergillosis and 1,837,272 (18.5%) cases of chronic pulmonary aspergillosis; *Candida* spp. was estimated to cause 1,565,000 (63.6%) cases of bloodstream infections/invasive candidiasis; and *Cryptococcus* spp. was estimated to cause 194,000 (75.8%) cases of cryptococcal meningitis. According to Benedict et al. (2022), the U.S. economic burden of fungal diseases was estimated at $11.5 billion in 2019, consisting of $7.5 billion for direct medical costs, $870 million for absenteeism-related productivity loss, and $3.2 billion for premature deaths. Notably, this value is likely underestimated due to underdiagnosis and underreporting.

On October 25, 2022, WHO released the first fungal priority pathogens list (FPPL), which flagged the most significant fungal threats to humans, with the major goals of guiding and coordinating global research and public health interventions and policies (WHO 2022). The FPPL classified the priority fungal pathogens into three groups based on their clinical significance: critical, high, and medium priority. The critical priority group includes *Cryptococcus neoformans*, *Candidozyma auris* (syn. *Candida auris*), *Aspergillus fumigatus*, and *Candida albicans*. The high priority group includes *Nakaseomyces glabrata* (syn. *Candida glabrata*), *Histoplasma* spp., Eumycetoma causative agents, Mucorales, *Fusarium* spp., *Candida tropicalis*, and *Candida parapsilosis*. The medium priority group includes *Scedosporium* spp., *Lomentospora prolificans*, *Coccidioides* spp., *Pichia kudriavzevii* (*Candida krusei*), *Cryptococcus gattii*, *Talaromyces marneffei*, *Pneumocystis jirovecii*, and *Paracoccidioides* spp. By prioritizing these pathogens, the FPPL aimed to guide global efforts to bridge knowledge gaps, improve surveillance, promote innovation in diagnostics and treatment, and alleviate the growing burden of antifungal resistance.

Over the years, China’s National Health Commission, national-level research institutes, and researchers have actively responded to the WHO’s call for increased attention to fungal diseases, including the 2022 WHO FPPL, contributing to global research and mitigation strategies against fungal infections. Some of those efforts are documented in peer-reviewed scientific publications that can be retrieved for analyses. Here, we analysed publication data from Chinese researchers in two databases: the Web of Science Core Collection (WOSCC) and the China National Knowledge Infrastructure (CNKI). WOSCC is a global citation and indexing database of publications run by Clarivate, while CNKI is China’s largest academic literature database. In this review, we focused on both WOSCC and CNKI publications to examine research on fungal diseases in China, the contribution made by Chinese researchers on the four critical priority and a few high and medium priority fungal pathogens, antifungal drug resistance and new drug developments, and China’s strategies, policies, and future directions.

## 2. Bibliographic methods

**Figure 1.**
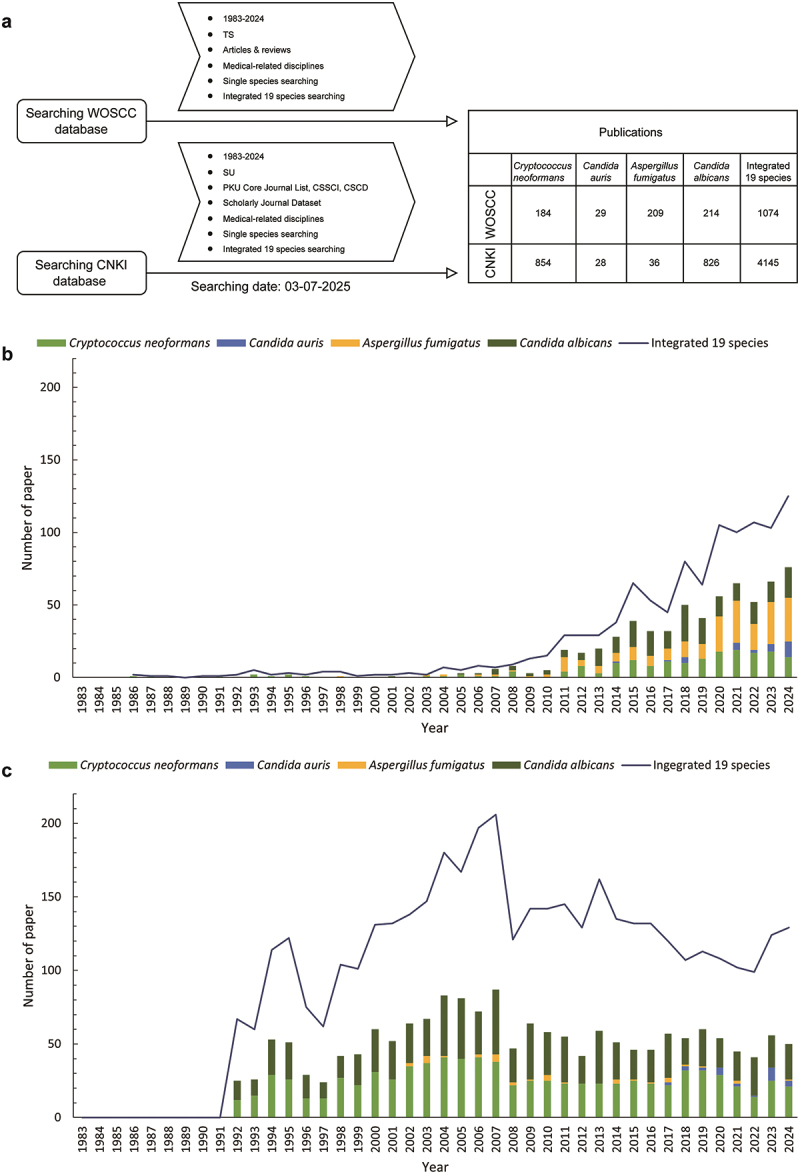
Overview of data screening and temporal trends in publication volume. (a) Data screening criteria and results. (b) Publication trends of critical group (*Cryptococcus neoformans*, *Candida auris*, *Aspergillus fumigatus*, *Candida albicans*) and 19 integrated species of WHO fungal priority pathogens in the WOSCC database from 1983 to 2024. (c) Publication trends of critical group (*Cryptococcus neoformans*, *Candida auris*, *Aspergillus fumigatus*, *Candida albicans*) and 19 integrated species of WHO fungal priority pathogens in the CNKI core academic journal dataset from 1983 to 2024.

To identify peer-reviewed publications on human fungal pathogens associated with China, a literature search was performed on July 3, 2025, using the WOSCC database (https://webofscience.clarivate.cn/wos/woscc/basic-search) representing publications in English language and the CNKI database (https://kns.cnki.net/kns8s/AdvSearch?classid=YSTT4HG0) representing publications in Chinese language (Figure 1a). For CNKI searches, we focused on publications on the Peking University (PKU) Core journal list, Chinese Social Sciences Citation Index (CSSCI), and Chinese Science Citation Database (CSCD) to maintain high publication standards. Searches were conducted using field tags such as TS (topic) for WOSCC and SU (subject category) for CNKI. The timeframe for the publications was set as 1983–2024. The search strategy consists of two parts applied to both the WOSCC and the CNKI databases: separate searches for the four critical priority fungal pathogens and integrated search for all pathogens on the FPPL.

For searches on the four critical priority pathogens, the search terms were: 1. *Cryptococcus neoformans* OR *Cryptococcus deneoformans* OR *Filobasidiella neoformans* OR Cryptococcosis OR Cryptococcal meningitis; 2. *Candida auris* OR *Candidozyma auris* OR Candidiasis OR Candidosis OR Candidemia OR yeast infection; 3. *Aspergillus fumigatus* OR Aspergillosis; 4. *Candida albicans* OR Candidiasis OR Candidosis OR Candidemia OR yeast infection.

For the integrated search, the search term was: *Cryptococcus neoformans* OR *Cryptococcus deneoformans* OR *Filobasidiella neoformans* OR *Cryptococcosis* OR *Cryptococcal meningitis* OR *Candida auris* OR *Candidozyma auris* OR *Aspergillus fumigatus* OR Aspergillosis OR *Candida albicans* OR Candidiasis OR Candidosis OR Candidemia OR yeast infection OR *Nakaseomyces glabrata* OR *Candida glabrata* OR *Histoplasma* spp. OR *Histoplasmosis* OR *Histoplasma capsulatum* OR *Eumycetoma* OR *Scedosporium boydii* OR *Pseudoalleschia boydii* OR *Madurella mycetomatis* OR *Trematospheria grisea* OR *Madurella grisea* OR Mucorales OR Mucormycosis OR *Rhizopus oryzae* OR *Rhizopus delemar* OR *Muc* OR Circinelloides OR *Lichtheimia corymbifera* OR *Lichtheimia ramosa* OR *Lichtheimia ornate* OR *Fusarium* spp. OR *Fusariosis* OR *Fusarium solani* OR *Fusarium oxysporum* OR *Fusarium fujikuroi* OR *Fusarium incarnatum-equiseti* OR *Fusarium clamydosporum* OR *Fusarium dimerum* OR *Fusarium sambucinum* OR *Fusarium concolor* OR *Fusarium lateritium* OR *Candida tropicalis* OR *Candida parapsilosis* OR *Candida metapsillosis* OR *Candida orthopsillosis* OR *Scedosporium* spp. OR *Scedosporiasis* OR *Scedosporium apiospermum* OR *Scedosporium aurantiacum* OR *Lomentospora prolificans* OR *Scedosporium prolificans* OR *Coccidioides* spp. OR *Cryptococcus gattii* OR *Talaromyces marneffei* OR *Penicillium marneffei* OR Talaromycosis OR Penicillosis OR *Pneumocystis jiroveci* OR *Pneumocystis pneumonia* OR *Pneumocystis carinii* OR *Paracoccidioides* spp.

All records were first retrieved with fungal keywords, then manually screened to retain only publications dealing with medical mycology; studies of fungi exclusively in environmental, agricultural, or industrial settings were excluded. Bibliometric analysis was conducted using VOSviewer (1.6.19) software and Excel (2024). For better visualization, entities (i.e. author, institute, keyword, or country) were included in the plots only if they surpassed the following thresholds. For WOSCC data, the thresholds were 20 publications for an individual institute, 10 publications for an individual author, keyword, and 5 publications for an individual country. For CNKI data, only plots regarding authors and keywords were applicable, with the thresholds of 10 publications for an individual author and 25 publications for an individual keyword.

## 3. Literature overview and trends

### 3.1. Overview of Chinese publications and volume changes over time

Our search strategy identified 5,219 articles published between 1983–2024, with 1,074 from WOSCC and 4,145 from CNKI. Figure 1 displays the searching strategy, publication distribution across pathogens per source, and yearly publication output stratified by pathogens. The detailed publication numbers in both databases in each year are shown in Supplementary Table S1.

The data show a rapid growth of annual publication volume in WOSCC after 2000 (Figure 1b). CNKI draws from the PKU core, CSSCI, and CSCD lists, so no data exist before 1991. The number experienced rapid increase afterwards with some fluctuations (Figure 1c), reaching the highest level so far in 2007 and then declined and stabilized with some fluctuations in CNKI. This time point (2007) coincides with the period when Chinese publications in the WOSCC database began to take off.

Over the study period, publications related to the four critical pathogens accounted for 59.2% and 42.1% of the WOSCC and CNKI records respectively (Figure 1a). *C. albicans* and *C. neoformans* have dominated the CNKI fungal pathogen publication output over the years, while publications on *C. auris* emerged only recently. This was not surprising because *C. auris* was described only in 2009 as a new species. In WOSCC publications, *C. auris* exhibits an upward trend in yearly output. Among the four critical fungal pathogens, *C. albicans* has the highest total number of publications, with 826 in CNKI and 214 in WOSCC, followed closely by *C. neoformans*, with 854 in CNKI and 184 in WOSCC. *A. fumigatus* ranked third (WOSCC: 209, CNKI: 36) and *C. auris* had the lowest number of publications in both databases (29, 28).

**Figure 2.**
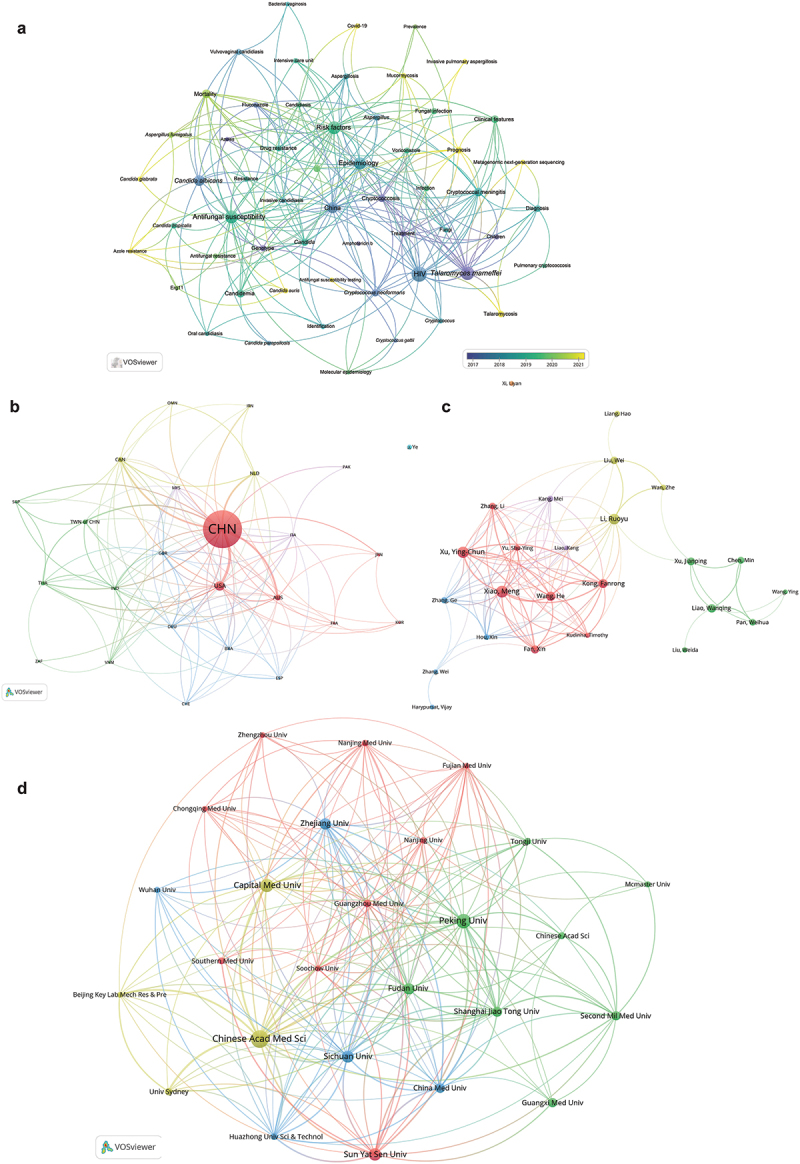
Co-occurrence analysis of 19 integrated species of WHO fungal priority pathogens in the WOSCC database from 1983 to 2024. (a) Keyword clustering map (threshold ≥ 10). Nodes represent keywords, and larger circles indicate higher frequency. Different colors represent different clusters. Distance between circles indicates the correlation between keywords; closer distance means higher correlation. (b) Global research collaboration (threshold ≥ 5). Each node represents a country or region. Larger nodes indicate higher publication output. More edges between countries or regions signify stronger cooperation levels. Different colored edges represent different collaboration networks. (c) Author collaboration relationships (threshold ≥ 10). Nodes represent authors, and larger nodes indicate higher publication output. Edges between nodes indicate co-authorship frequency, with more edges suggesting higher collaboration intensity. Different colored edges represent different author collaboration networks. (d) Organizational collaboration network (threshold ≥ 20). Nodes represent organizations; larger nodes indicate higher publication output. Edges represent collaborative relationships between organization; more edges signify stronger collaboration. Different colored edges represent different organizational collaboration networks. Geographic abbreviations of Figure 2b: Australia: AUS; Brazil: BRA; Canada: CAN; Great Britain: GBR; France: FRA; Germany: DEU; India: IND; Iran: IRN; Italy: ITA; Japan: JPN; Malaysia: MYS; Netherlands: NLD; Oman: OMN; Pakistan: PAK; People’s R China: CHN; Singapore: SGP; South Africa: ZAF; South Korea: KOR; Spain: ESP; Switzerland: CHE; Taiwan of China: TWN of CHN; Thailand: THA; USA: USA; Vietnam: VNM.

**Figure 3.**
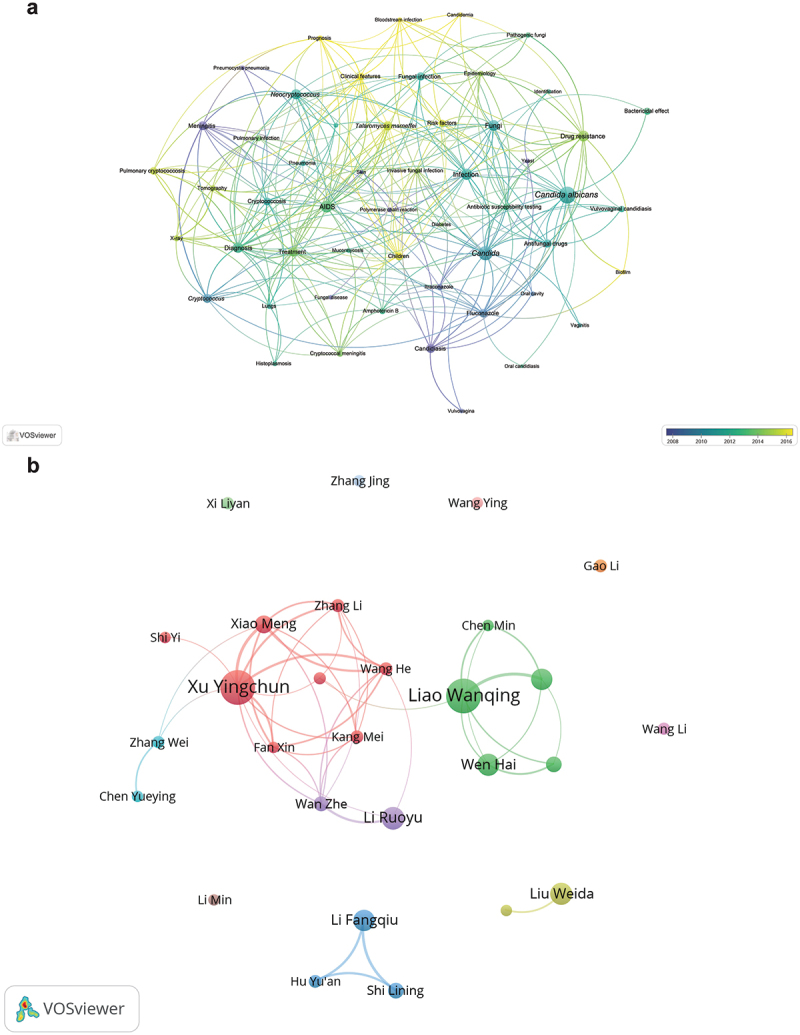
Co-occurrence analysis of 19 pathogens on WHO fungal priority pathogens list in the CNKI core academic journal dataset from 1983 to 2024. (a) Keyword clustering map (threshold ≥ 25). Nodes represent keywords, and larger circles indicate higher frequency. Different colors represent different clusters. Distance between circles indicates the correlation between keywords; closer distance means higher correlation. (b) Author collaboration relationships (threshold ≥ 10). Nodes represent authors, and larger nodes indicate higher publication output. Edges between nodes indicate co-authorship frequency, with more edges suggesting higher collaboration intensity. Different colored edges represent different author collaboration networks.

**Figure 4.**
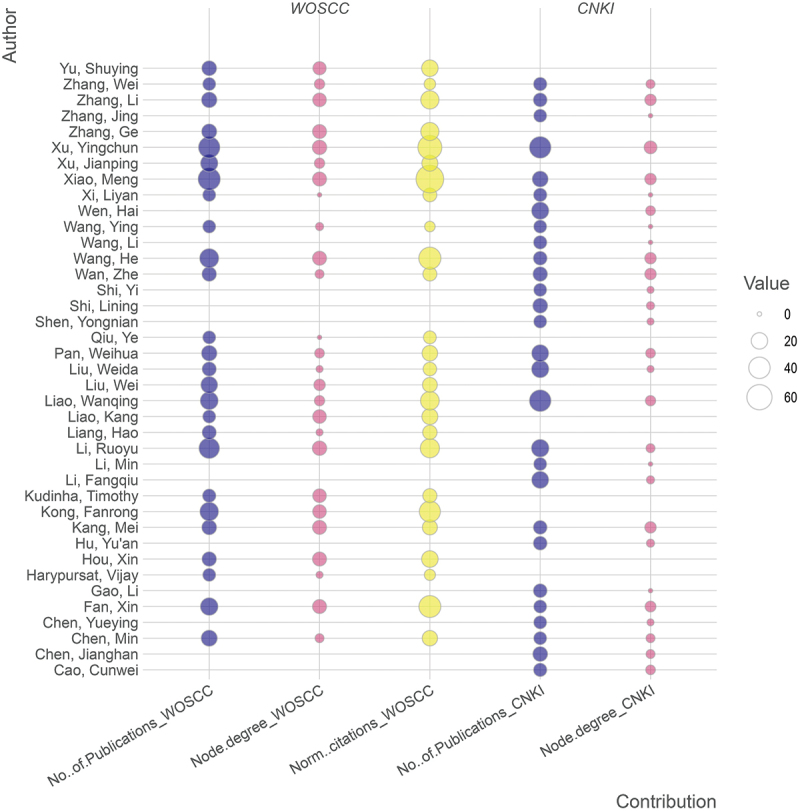
Bubble plot showing authors’ contributions based on WOSCC and CNKI records. A threshold ≥ 10 in at least one of the categories within each database was used to select authors for inclusion in the two respective databases. No. of publications: total number of documents authored by that person. Node degree: number of co-authored publications. Norm. citations: the average number of citations per publication.

Based on the extracted bibliographic information, in the sections below, we describe and display (i) the key scientific themes based on keywords; (ii) top 10 journals in each database where the medical mycology findings were reported; (iii) top publishing institutions; (iv) top publishing authors; and (v) the main countries with researchers who Chinese investigators collaborated with. To select the threshold values for optimal visual displays, we set different items in each figure and selected two to three thresholds for analyses. The threshold analyses results are shown in Supplementary Table S2 where the highlighted numbers in bold were the selected thresholds for displays in Figures 2, 3, and 4.

### 3.2. Overview of key bibliometric indicators

#### 3.2.1. Core scientific themes among publications

Keyword analyses revealed a high diversity of topics of medical mycology research (Figures 2a and 3a; Supplementary Table S3). Specifically, the WOSCC publications revolved around topics such as HIV, antifungal susceptibility, risk factors, epidemiology, and *C. albicans*. HIV is an important topic in mycology research as patients with HIV have weakened immune systems, making them highly susceptible to fungal infections and more likely to die. Understanding the interaction between HIV and fungal infections can help improve diagnosis, prevention, and treatment. Besides HIV, there are many other risk factors for developing fungal diseases, which include but not limited to cancers, diabetes, and organ transplantation. Recognizing risk factors is essential to predict risk populations, apply early intervention, and/or prophylactic treatment. Antifungal susceptibility is an inescapable issue in fungal research. There is a growing problem of antifungal resistance in fungal pathogens such as *C. auris* and *A. fumigatus*. Identifying susceptibility patterns and resistance mechanisms can help inform effective treatment, track drug resistance trends, and develop new antifungal agents. Epidemiological research helps understand incidence, prevalence, and mortality of fungal infections both globally and regionally, which is critical for public health planning, particularly in resource-limited areas where such infections are underdiagnosed and underreported. *C. albicans* has been known since 400 BC, when it was found to cause an infection called “thrush” (Anderson and Odds 1985). Large-scale research began to increase significantly starting in the mid to late 20^th^ century, due to the development of molecular biology and rising clinical importance of fungal pathogens (Kabir et al. 2012). Given the long-standing recognition as a human pathogen and clinical significance, *C. albicans* holds a central place in medical mycology.

As expected, the dominant themes of CNKI publications largely overlap with those in WOSCC, including *C. albicans*, HIV/AIDS, and drug resistance. In addition, these scientific themes maps also revealed several emerging research topics (highlighted with yellow dots in Figures 2a and 3a), such as metagenomic next-generation sequencing, genomics, *C. auris*, Covid-19, Talaromycosis, *Talaromyces marneffei*, and antifungal susceptibility, all with an increasing number of publications since 2021. These new focused areas pinpoint the most recent advances and/or hot topics in medical mycology research in China. Together, these topics are connected with each other and with other related ones, forming an increasingly integrated knowledge landscape of medical mycology research.

#### 3.2.2. Top publishing journals in WOSCC and CNKI for Chinese medical mycology

The overall top ten English publishing journals on Chinese medical mycology research from 1983 to 2024 in the WOSCC database were BMC Infectious Diseases (65 publications, 6.1% of the total), Mycopathologia (51, 4.7%), Infection and Drug Resistance (50, 4.7%), Frontiers in Microbiology (39, 3.6%), Mycoses (39, 3.6%), Medical Mycology (28, 2.6%), Frontiers in Cellular and Infection Microbiology (26, 2.4%), Chinese Medical Journal (25, 2.3%), PLOS ONE (21, 2.0%), and Journal of Clinical Microbiology (18, 1.7%). These international English language journals are also the common platforms for medical mycology researchers from both developed and other developing countries.

The overall top ten Chinese publishing journals on Chinese medical mycology research in the CNKI database are Zhongguo Zhenjunxue Zazhi (literal translation “Chinese Mycology Journal”; 327, 7.9%), Zhonghua Yiyuanganranxue Zazhi (literal translation “Chinese Hospital Infections Journal”; 190, 4.6%), Zhongguo Pifuxingbingxue Zazhi (literal translation “Chinese Skin Diseases Journal”; 179, 4.3%), Chinese Journal of Dermatology (158, 3.8%), Journal of Clinical Dermatology (142, 3.4%), Chinese Journal of Practical Internal Medicine (105, 2.5%), Chinese Journal of Clinical Laboratory Science (101, 2.4%), Chinese Journal of Infection and Chemotherapy (94, 2.3%), Chinese Journal of Disinfection (88, 2.1%), Chinese Journal of Microecology (80, 1.9%).

These 20 journals are popular among Chinese researchers due to their focus on global or national medical mycology, interdisciplinary scope, and/or efficient publication processes. They serve as key platforms for research in fungal pathogenesis, infectious disease epidemiology, and antifungal resistance.

#### 3.2.3. Top publishing institutions in the WOSCC and CNKI databases

In the WOSCC database from 1983 to 2024, 26 institutions contributed 20 or more manuscripts each to medical mycology research in China (Supplementary Table S4). After merging duplicates from the same institution, the overall top ten Chinese institutions in the WOSCC database were Chinese Academy of Medical Sciences (125, 11.6%), Peking University (94, 8.8%), Capital Medical University (79, 7.4%), Sun Yat-Sen University (75, 7.0%), Sichuan University (71, 6.6%), Zhejiang University (62, 5.8%), Fudan University (61, 5.7%), Shanghai Jiao Tong University (51, 4.7%), China Medical University (49, 4.6%), and PLA Second Military Medical University and Guangxi Medical University (both 46, 4.3%). Among them, Peking University, Chinese Academy of Medical Sciences, and Fudan University have the highest levels of inter-institutional collaborations with other institutes (Figure 2d).

The ten most prolific Chinese institutions that published medical mycology research in Chinese language journals as revealed by the CNKI database from 1991 to 2024 were Shanghai Changzheng Hospital (151, 3.6%), Peking University First Hospital (73, 1.8%), Nanjing General Hospital of Nanjing Military Region (64, 1.5%), People’s Liberation Army General Hospital (55, 1.3%), West China Hospital, Sichuan University (50, 1.2%), The First Affiliated Hospital of Guangxi Medical University (49, 1.2%), Huashan Hospital Affiliated to Fudan University (43, 1.0%), The First Affiliated Hospital of Chongqing Medical University (40, 1.0%), Peking Union Medical College Hospital (34, 0.8%), and The Third Affiliated Hospital of Sun Yat-sen University (32, 0.8%).

As shown above, the top Chinese institutions who have published medical mycology research in English language journals in the WOSCC database were primarily from academic institutions such as dedicated research institutions and universities. In contrast, the top institutions who have published medical mycology research in Chinese language journals in the CNKI database were primarily from hospitals affiliated with medical universities.

#### 3.2.4. Top publishing authors and their co-authorship relationships in the WOSCC & CNKI databases

The details of authors with more than 10 publications in either database are shown in Supplementary Table S5. Figures 2c and 3b display authors’ publication counts and the co-authorship relationships based on WOSCC and CNKI records respectively. Some researchers have a high number of contributions in both WOSCC and CNKI databases, helping bridge international and regional scholarly communications and fostering greater knowledge exchange and collaboration (Figure 4).

The top publishing authors with > 20 publications each in medical mycology in the WOSCC database include Meng Xiao (44 publications, 13 collaborations), Yingchun Xu (40, 14), Ruoyu Li (36, 14), He Wang (30, 13), Fanrong Kong (28, 12), Wanqing Liao (25, 5), Xin Fan (24, 13), and Jianping Xu (23, 5). Among them, research collaborations were frequent among Meng Xiao, Yingchun Xu, He Wang, Fanrong Kong, and Xin Fan.

In the CNKI database, the top publishing authors with > 20 publications each were Yingchun Xu (41, 10), Wanqing Liao (41, 5), Ruoyu Li (24, 3), Weida Liu (23, 1), Hai Wen (23, 4), Fangqiu Li (22, 2), and Weihua Pan (22, 4). Compared to those in the WOSCC database, collaborations were less frequent among the top CNKI contributors in medical mycology.

#### 3.2.5. Temporal shift toward international visibility and collaboration

The WOSCC database showed an upward trend of publishing English-language papers among Chinese researchers, especially after 2007, indicating an increasing international presence (Figure 1b). In addition, based on the WOSCC records, Chinese researchers have been frequently collaborating with those from the United States, Australia, Canada, the Netherlands, Thailand, India, England, Japan, Brazil, and Germany (Figure 2b; Supplementary Table S6). Indeed, a number of institutions in China and other countries have established close research collaborations (Figure 2d). For example, the University of Sydney has co-authored numerous studies with many Chinese institutions including Chinese Academy of Medical Sciences, Huazhong University of Science and Technology, Sun Yat-sen University, Sichuan University, Capital Medical University, and Peking University.

## 4. Critical priority fungal pathogens and China’s research advances

After the publication of the first FPPL in 2022, research on WOSCC’s 19 fungal pathogens in China reached 125 papers in 2024, its highest ever (Supplementary Table S1). Similarly, the number of papers on the four critical priority fungal pathogens was also the highest in 2024 in the WOSCC database. In the CNKI database, the number of publications on the 19 pathogens on FPPL dropped between 2018–2022 but then rebounded to exceeding 120 papers in both 2023 and 2024 (Supplementary Table S1). The increases reflect the Chinese research community’s overall output as well as their prompt response to the new WHO call for actions. In this section, we describe WHO’s four critical priority fungal pathogens and the advances made by Chinese researchers since 2022.

### 4.1. Cryptococcus neoformans

#### 4.1.1. Epidemiology and molecular characteristics

*Cryptococcus neoformans* (*C. neoformans*) is a globally distributed encapsulated pathogenic yeast, commonly found in natural environments such as soil, bird droppings, and decaying wood. It can cause various infections, including fungemia, pneumonia, and meningitis. Cryptococcal infections (cryptococcosis) are established after inhalation of airborne spores or yeast cells, primarily affecting the lung area. In healthy individuals, the fungus is typically cleared by the immune system. However, when the immune system is compromised, *C. neoformans* can disseminate through the bloodstream to infect the brain and other body parts. Patients with weakened immune systems are the primary risk group for cryptococcosis, especially organ transplant recipients, patients with AIDS, and hematological malignancies (Dao et al. 2024). The mortality rate of *C. neoformans* infections is estimated between 41% and 61%, with cryptococcal meningitis reaching as high as 90% (Rathore et al. 2022; Dao et al. 2024). While the median survival of people living with AIDS after cryptococcal diagnosis was estimated to be 244 days, factors such as the infection severity, patient’s immune status, and proper treatment availability can significantly affect the overall survival and prognosis (Hevey et al. 2019).

*C*. *neoformans* is a haploid species with a genome size of approximately 18.5–20 million base pairs. It is a member of a species complex which originally included several genetically distinct lineages. Using rabbit antisera against capsular polysaccharide, the species complex was initially classified into multiple serotypes, namely *C. neoformans* var. *grubii* (serotype A), *C. neoformans* var. *neoformans* (serotype D), *C. gattii* (serotypes B and C), and hybrids (serotypes AD, AB, etc.) (Kwon-Chung and Varma 2006; Okabayashi et al. 2006; Hitchcock and Xu 2023). These serotypes are mostly distinguishable at genomic level and have each been further classified into subgroups based on molecular types: *C. gattii* (molecular types VGI, VGII, VGIII, VGIV, and VGV), *C. neoformans* var. *grubii* (molecular types VNI, VNII, and VNB), *C. neoformans* var. *neoformans* (molecular type VNIV), serotype AD hybrids (VNIII), and hybrids between *C. gattii* and *C. neoformans* variants. Due to their genetic, ecological, and clinical differences among the molecular types, *C. gattii* is now reclassified as a separate species complex. Though there have been debates, the four molecular types VGII–VGIV and VGIV/VGIIIc of *C. gattii* species complex have been proposed for four new species *C. bacillisporus*, *C. deuterogattii*, *C. tetragattii*, and *C. decagattii* (Kwon-Chung and Varma 2006; Hagen et al. 2015; Yamamura and Xu 2021). *C. neoformans* primarily infects immunosuppressed, while *C. gattii* are more often reported in immunocompetent individuals (Chen et al. 2014; Beardsley et al. 2019). Moreover, *C. gattii* is mainly found in tropical and subtropical areas, unlike the widespread distribution of *C. neoformans*. In fact, *C. neoformans* var. *grubii* is responsible for most cases of cryptococcosis, accounting for 95% (Kwon-Chung et al. 2015).

#### 4.1.2. *Phenotypic features, diagnosis, and treatment of* C. neoformans

*Cryptococcus neoformans* exists as a budding yeast with a size of 4–6 µm in diameter. This species primarily reproduces asexually through budding but can also reproduce through bisexual and unisexual mating (Zhao and Lin 2021; Hitchcock and Xu 2022). *C. neoformans* can grow at temperatures ranging from 20 °C to over 40 °C, with an optimal growth temperature of 30 °C (Heere et al. 1975). On Sabouraud dextrose agar, *C. neoformans* forms smooth, translucent, and whitish colonies, while on bird seed agar, *C. neoformans* produces reddish-brown to black colonies.

Diagnosis of *C. neoformans* infections involves a combination of methods such as direct microscopy, culture, antigen detection, molecular detection, and imaging (Perfect and Bicanic 2014; Yamamura and Xu 2021; Cleveland Clinic 2023). Direct microscopy, particularly with India ink stain, is a quick method to examine the encapsulated yeast cells in cerebrospinal fluid (CSF) and other body fluids. Antigen test is more accurate than microscopy, which detects capsular polysaccharide antigen in CSF or serum. Cultures from CSF, blood, or respiratory samples are more reliable diagnosis option but take longer time. Molecular methods such as PCR and sequencing can aid in species- and strain-level and antifungal gene identification. Computed tomography, chest X-rays, and magnetic resonance imaging can locate the sites of infection and assess the scale and severity of infection (Perfect and Bicanic 2014; Yamamura and Xu 2021; Cleveland Clinic 2023).

Various treatment strategies are available against cryptococcosis and other fungal infections. Among them, antifungal therapy is the most important, particularly in invasive and life-threatening cases. The main antifungal drug classes include azoles, polyenes, and echinocandins (Fisher et al. 2022). Azoles target the enzyme lanosterol 14α-demethylase to inhibit ergosterol synthesis, thereby disrupting cell membrane integrity and function. Polyenes bind to ergosterol directly, causing damages to the pathogen cell membrane. Echinocandins inhibit β-1,3-glucan synthesis, weakening the fungal cell walls (Cortegiani et al. 2018). Another effective antifungal agent is 5-fluorocytosine, which inhibits synthesis of both DNA and RNA by incorporating into fungal RNA (Padda and Parmar 2024).

For non-severe cryptococcosis, fluconazole is routinely used, but in severe cases and those with fluconazole resistance, a combination of amphotericin B and 5-fluorocytosine is required. Surgery may be performed to remove large fungal masses or relieve intracranial pressure on the brain. For AIDS patients, it is also recommended to commence antiretroviral therapy to reconstruct immune competence in patients with cryptococcal meningitis after antifungal treatment (Perfect and Bicanic 2014).

#### 4.1.3. Virulence factors

*Cryptococcus neoformans* employs various factors to establish diseases in patients with impaired immune functions: capsule, melanin, biofilm, mannitol, and phospholipase. These virulence factors are described below.

The cryptococcal capsule is a polysaccharide layer surrounding the cell wall and is mainly composed of glucoronoxylomannan and galactoxylomannan. Capsule is a major virulence factor in *C. neoformans*, and its production is significantly higher during infection. The capsule can shield the antigenic components on the fungal cell surface from the host immune system (Vecchiarelli et al. 1996) and restricts phagocytosis by macrophages, monocytes, and neutrophils (Chun et al. 2011). Study has shown that capsule formation and thickness are influenced by various genetic and environmental conditions (Vogan et al. 2016). For example, lower level of glucose might affect the expression of capsule-associated genes (*CAP* genes) *in vitro* thereby accelerating the production of capsular components (Okabayashi et al. 2005). Meanwhile, magnesium ions can induce capsule formation via the upregulation of *CAP* genes (Rathore et al. 2016). Mannitol treatment was also found to result in larger capsules in *C. neoformans* and reduced brain dissemination in mice (Guimarães et al. 2010).

Melanin is another major virulence factor which contributes to cryptococcal cell survival against attacks by the host immune system and damages by oxidative stress, antifungal stress, ultraviolet light, and ionizing radiation. Melanin reduces inflammatory cytokine production, likely through modulating inflammatory signaling pathways (Guan et al. 2021), although in certain contexts, it promotes inflammation through other pathways (Gasque and Jaffar-Bandjee 2015). Melanin can suppress chemokine secretion by airway epithelial cells, resulting in impaired immune cell trafficking to the site of infection (Reedy et al. 2024). Additionally, melanin has been shown to inhibit phagosome maturation by blocking LC3-associated phagocytosis (Akoumianaki et al. 2016). Moreover, melanin reduces the generation of reactive oxygen species in phagocytes, thereby promoting immune evasion and enhancing survival within the host (Sarangarajan and Apte 2005).

Similar to the protective effects of capsule and melanin, biofilms can also protect *C. neoformans* cells from antifungals and macrophage phagocytosis in the host and likely enhance their survival in natural environments (Benaducci et al. 2016; Aslanyan et al. 2017). Biofilm formation in *C. neoformans* is influenced by several factors, including capsule production, surface characteristics, and environmental cues. Since cryptococcus capsule and cryptococcal biofilm exopolymeric matrix have the same major component glucuronoxylomannan, formation of biofilm is also associated with capsule production. It was reported that *C. neoformans* strain C536 which has the capsular *CAP59* gene deletion cannot generate biofilm, suggesting the necessity of capsular polysaccharide for biofilm formation (Martinez and Casadevall 2005). Surface properties are another factor affecting *C. neoformans* biofilm formation, with materials such as polyvinyl and those coated with CSF or antibodies exhibiting enhanced adhesion and biofilm development (Martinez and Casadevall 2015). *C. neoformans* can form robust biofilms under environmental conditions (25–30 °C, neutral pH, ambient CO₂) and in the presence of carbon sources such as glucose or mannose (Martinez and Casadevall 2007).

Intracellular synthesis and accumulation of mannitol contribute to the pathogenesis and survival of *C. neoformans* in the host by enhancing its resistance to various stresses, including osmotic stress, heat stress, and damage caused by reactive oxygen intermediates (Buchanan and Murphy 1998). Another virulence factor in *C. neoformans* is the enzyme group phospholipases that break down phospholipids in cell membrane, allowing pathogens to invade and cause infections in host cells. For example, study has shown that disruption of the phospholipase B1 coding gene *PLB1* reduces the virulence of *C. neoformans* in animal models of cryptococcosis (Cox et al. 2001).

#### 4.1.4. Research advances in China since 2022

Since 2022, Chinese scientists have conducted multiple epidemiological studies to identify cryptococcosis characteristics, including the distribution, predominant genotype(s), and at-risk populations in various regions within and sometimes outside of China (Zhang et al. 2022, 2023; Zhou et al. 2022b; Bilal et al. 2023c; Chen et al. 2023a; Tian et al. 2023; Zhu et al. 2023; Tao et al. 2024; Wang et al. 2024a, 2024c). Although these studies cover data across different geographic areas in the past two decades, several consistent patterns have been observed. Overall, males are more prone to cryptococcosis compared to females and the multilocus sequence type (ST) 5 (VNI) was found to be the most dominant among the isolated clinical strains in China. In addition, *C. neoformans* strains exhibited decreased susceptibility to antifungal drugs over the years. For example, Tian et al. (2023) analyzed 414 strains isolated from nationwide patients admitted to Huashan Hospital in Shanghai during 2005–2021. Although a low frequency (less than 10%) of the isolates had non-wild-type minimum inhibitory concentration (MIC) against antifungal drugs, a gradual increase trend was identified for ST5 strains when stratifying the antifungal geometric mean MICs over time. Wang et al. (2024a) analyzed pathogenic fungi collected from 54 hospitals in Shandong province during 2018–2021 and showed that 16.67% of the isolated *C. neoformans* strains had the non-wild-type phenotype against amphotericin B. Besides, Bilal et al. (2023c) conducted a six-year retrospective study which revealed a dramatic increase in the number of infection cases during the study. Other analyses took a more in-depth approach by stratifying samples to uncover subgroup-specific patterns and risk factors. For instance, Tao et al. (2024) grouped cryptococcal patients into the pulmonary infection group and extrapulmonary infection group and conducted regression analysis to investigate the risk factors for infection dissemination. They found that disseminated infections were likely associated with high levels of neutrophils and neutrophil-to-lymphocyte ratio, and low levels of lymphocytes and monocytes. Liu et al. (2023a) explored the clinical characteristics and prognostic factors of AIDS patients with cryptococcosis, comparing those with and without cryptococcal meningitis (CM). Their study identified several prognostic risk factors: age, headache, nuchal rigidity, first intracranial pressure elevation, CD4/CD8, days in the hospital, and CSF protein level. The CM group exhibited higher positive rates of cryptococcal capsular antigen, India ink staining and culture of the CSF, as well as a higher incidence of neurological symptoms, such as headache, impaired consciousness, nuchal rigidity, elevated initial intracranial pressure, and increased mortality. Overall, these studies identify both shared and unique features of cryptococcal infections in Chinese patients, results that could promote timely intervention in risk groups.

Early and accurate diagnosis with timely intervention is critical in reducing the cryptococcosis burden and improving patients’ outcomes. To date, the IMMY CrAg® LFA (lateral flow assay) is the leading antigen test for cryptococcal diagnosis due to its rapid performance, excellent diagnostic accuracy, and ease of use. Cryptococcal capsular polysaccharide detection K-set LFA (FungiXpert) was developed in China as its counterpart. According to a study evaluating 199 samples, the FungiXpert LFA demonstrated performance comparable to the IMMY CrAg LFA, with some potential advantages in semi-quantitative sensitivity, highlighting its use as an alternative screening method of cryptococcosis (Liu et al. 2022). Besides, PCR amplification techniques are also crucial tools for diagnosing cryptococcosis. A recently developed novel MME-18 (multiplex PCR detection for 18 pathogens of meningitis and encephalitis) successfully identified 96.3% (79/82) of cases, indicating high reliability in clinical settings (Si et al. 2022). Moreover, metagenomic next-generation sequencing demonstrated perfect specificity in detecting *C. neoformans* from the CSF of AIDS patients, establishing it as a highly reliable diagnostic tool (Zhu et al. 2022). Despite these advances, continuing efforts are needed to explore new approaches to make diagnosis faster, more specific, highly sensitive, and more comprehensive.

Over the years, Chinese research groups have screened and evaluated the anti-microbial activity of a wide range of compounds derived from natural sources to identify potential new antifungal agents. Several substances have been identified to possess anti-*C. neoformans* activity, including peptide QS18 from the venom gland of *Chilobrachys liboensis* (Michira et al. 2024), quinomycin A metabolized by endophytic microbes in medicinal plants (Yang et al. 2023), and norsesquiterpenoids from soil-derived *Streptomyces* (Cao et al. 2024). In addition, researchers have found anti-*C. neoformans* IgG antibodies in intravenous immunoglobulin, underlining the potential application of intravenous immunoglobulin in therapeutic settings. To date, antifungal drugs remain the standard first-line treatment for cryptococcosis. Although widespread drug resistance has not been observed in *C. neoformans*, developing new drugs and treatment strategies are important to lower costs, improve patient outcomes, and mitigate the potential for future resistance in this critical human fungal pathogen.

### 4.2. Candidozyma auris

#### 4.2.1. Epidemiology and molecular characteristics

*Candidozyma auris* (*C. auris*, syn. *Candida auris*) is a recently emerged human fungal pathogen posing a global health threat. This pathogen has drawn significant public attention due to its multidrug resistance, persistence in clinical settings, and rapid transmission dynamics at diverse scales. The species was initially isolated from the ear canal of a Japanese patient in 2008 (Satoh et al. 2009). Since then, infection cases have been reported across the globe at an accelerating and concerning rate (Chow et al. 2020; Kohlenberg et al. 2022). For example, by the end of 2023, there had been 10,788 cases reported from the United States (CDC 2024). *C. auris* can colonize and infect multiple body sites including the skin, the gastrointestinal tract, the genitourinary tract, the respiratory tract, etc. Invasive infections can also occur, especially in critically ill patients with mechanical ventilation and invasive devices. It is estimated that the pooled mortality rates for patients infected by *C. auris* is as high as 39% (Chen et al. 2020).

*C*. *auris* has a haploid genome of around 12.4 million base pairs. Genomic analyses identified six distinct clades within the species, exhibiting geographic variations among the phylogenetic clades. Specifically, clade I were mainly identified in South Asia, II in East Asia, III in South Africa, IV in North America, V in Iran, and VI in Singapore (Chow et al. 2019, 2020; Suphavilai et al. 2023). There is a trend of multiple clades being successively discovered from the same geographic regions (Chow et al. 2020). However, most continents and many countries now have strains representing two or more clades (Wang and Xu 2022, 2024). The co-occurrence of multiple clades and rapid spread are attributed to increasingly frequent global travel, international trade and commerce, and utilization of globalized healthcare system.

#### 4.2.2. *Phenotypic features of* C. auris *and diagnosis and treatment of* C. auris *infections*

*C*. *auris* grows as oval to elongated yeast cells, measuring 2–5 µm in diameter. Under high salinity conditions and depletion of heat-shock proteins, *C. auris* can grow in pseudohyphae-like forms (Kim et al. 2019). *C. auris* undergoes asexual reproduction through budding, while sexual reproduction has been inferred but not observed yet (Chowdhary et al. 2013; Wang and Xu 2022). This fungus grows well at 37 °C and can also thrive at 42 °C (Sikora et al. 2023). On Sabouraud dextrose agar, *C. auris* forms smooth, cream to pink colonies, while on CHROMagar, colonies can exhibit various color morphologies, typically ranging from pale to dark pink (Cortegiani et al. 2018; Sikora et al. 2023).

In standard laboratory testing, *C. auris* can be easily misidentified as *C. haemulonii*, *C. lusitaniae*, etc., due to similar morphology and biochemical traits. Therefore, MALDI-TOF MS and molecular methods, such as PCR-based assays and DNA sequencing, are needed for accurate identification. The Vitek 2 system can also be used if neither of these techniques is available (Lockhart et al. 2022).

The primary treatment for *C. auris* infections is antifungal medication, particularly echinocandins. In cases with cross-resistance, a combination of antifungal drugs or other treatments may be needed. Study has shown that the removal of central catheters can improve the prognosis of patients with candidemia and lower Charlson Comorbidity Index scores (Lee et al. 2019).

#### 4.2.3. *Virulence factors and drug resistance in* C. auris

*C. auris* has multiple strategies to survive in host, including biofilm formation, thermotolerance, hydrolase production, immune evasion, and drug resistance.

*C. auris* can form biofilms on host tissues and medical surfaces, protecting cells from stressors and extending their persistence in the environment. Several genes involved in biofilm formation have been identified in *C. auris*. The cell wall glycoproteins coding gene *ALS4* that contributes to biofilm formation in *C. albicans* also played a role in *C. auris* biofilm formation, surface colonization, and virulence (Bing et al. 2023). Transcriptomic factor Ume6 promotes biofilm formation through regulating agglutin-like sequence adhesin Als4498, adhesin Scf1, and the hypha-specific G1 cyclin-related protein Hgc1 (Louvet et al. 2024). Other related genes are β-1,6- glucan synthase gene *KRE6*, cell-surface adhesin coding gene *ALS5*, and quorum-sensing molecules (Jakab et al. 2023; Fayed 2025).

*C. auris* can tolerate elevated temperatures up to 42 °C, allowing it to survive and adapt to the human body’s condition. It has been shown that the Ras/cAMP/PKA signaling pathway plays a critical role in *C. auris* thermotolerance, and deletion of *PDE2* or *BCY1* leads to hyperactivation of this pathway making *C. auris* cells thermosensitive and more vulnerable to nutrient limitation (Kim et al. 2023).

*C. auris* can produce secreted hydrolytic enzymes, aiding in tissue invasion and nutrient acquisition. For instance, aspartyl proteinases can degrade host proteins, phospholipases can break down phospholipids in host cell membranes, and lipases can hydrolyze lipids (Lockhart 2014; Rossato and Colombo 2018; de Jong and Hagen 2019).

Compared to *C. albicans*, *C. auris* has altered cell wall architecture with a lower level of β-glucans, making it less recognized and engulfed by macrophages and dendritic cells (Holt and Nett 2024). In addition, *C. auris* sepsis has been shown to induce an immune-suppressive phenotype through PD-1/PD-L1 induction, promoting immune escape (Wurster et al. 2022). Moreover, *C. auris* can induce macrophage death by depleting glucose and triggering metabolic stress in the host (Weerasinghe et al. 2023).

Over 90% of reported *C. auris* isolates are fluconazole resistant, with some variation across different clades and geographic distribution (Wang and Xu 2024). A substantial proportion is resistant to amphotericin B, while a small percentage is resistant to echinocandins (Ahmad and Alfouzan 2021; Wang and Xu 2024). Strikingly, strains that resistant to all three main antifungal classes have been reported, highlighting the urgent needs of developing new drugs and treatment strategies (Wang and Xu 2024). Several genes and mutations associated with drug resistance have been identified in *C. auris*. The most noteworthy gene conferring fluconazole resistance is *ERG11*, which encodes lanosterol 14-alpha-demethylase and is necessary for ergosterol biosynthesis. Three missense mutations in *ERG11* commonly detected in resistant strains are F126L, Y132F, and K143R (Chowdhary et al. 2018; Chaabane et al. 2019). Several candidate genes involved in exporting drugs out of the cell were also identified, including *CDR1* which encodes an ATP-binding cassette transporter, the transcription factor of *CDR1* encoded by *TAC1B*, and *MDR1* which encodes an integral membrane protein (Rybak et al. 2019, 2020; Yadav et al. 2021). Genetic basis for amphotericin B resistance in *C. auris* involves mutated *ERG2*, *ERG3*, *ERG5*, *ERG6*, and *ERG11* (Carolus et al. 2020, 2021; Kordalewska et al. 2021; Rybak et al. 2022). These mutated genes cause acquired amphotericin B resistance through altering the membrane permeability. Regarding echinocandins, resistance can result from mutations in the hotspot regions of *FKS1* (e.g., S639F, D642Y, R1354H, and R1354Y) (Chowdhary et al. 2018; Kordalewska et al. 2018; Ahmad and Alfouzan 2021). *FKS1* encodes β-1,3-glucan synthase, a key component of the fungal cell wall. In addition, genome-wide association studies based on the global samples identified many novel mutations associated with drug resistance in *C. auris* (Wang and Xu 2024).

#### 4.2.4. Research advances in China since 2022

Our database searches identified two studies describing *C. auris* burden in China. According to Bilal et al. (2022), 108 (0.24%) *C. auris* samples were identified among a total of 44,716 *Candida* isolates from mainland China between 2011 and 2021, and all of the 108 samples were from Northeast China. The first case of *C. auris* infection in southern China was found in 2023 when seven patients with candidemia were admitted to local hospitals in Guangdong (Peng et al. 2024). However, by the end of 2023, *C. auris* had spread to 10 provinces in China (Bing et al. 2024a).

Several studies by Chinese research groups analyzed antifungal resistance mechanisms. Bing et al. (2024a) carried out a nationwide retrospective study which included 312 *C. auris* cases from 2018 to 2023. They identified that these isolates belonged to three clades I–III, and almost all of them exhibited resistance to fluconazole (98.7%). However, only a small proportion were resistant to amphotericin B (4.2%) and caspofungin (2.2%). They found clade III isolates harbored the VF125AL mutation in *ERG11* and clade I fluconazole-resistant isolates had the Y1332F mutation in *ERG11*. While clade II fluconazole-resistant isolates had no *ERG11* mutations, they had the M653I mutation in *TAC1B*. Meanwhile, the S639F hotspot mutation of *FKS1* was identified in the isolates resistant to caspofungin. These findings suggest that distinct patterns of genetic mutations exist across the three clades, likely contributing to their drug resistance. A separate analysis by Yang et al. (2024) identified the A395T mutation in *ERG11* as the cause of reduced fluconazole susceptibility in strain CA01 through molecular docking. Chen et al. (2024c) uncovered several potential mutations related to *in vivo*-acquired drug resistance, including the Y132F mutation in *ERG11* and the A585S mutation in *TAC1B* for fluconazole resistance, the *F214L* mutation in *TAC1B* for voriconazole resistance, and a novel frameshift mutation in *SNG1* for amphotericin B resistance. Furthermore, Tian et al. (2024) integrated clinical, genomic, and transcriptomic data to explore the genomic mutations contributing to amphotericin B resistance. Briefly, they found strains with an elevated amphotericin B MIC harbored the T308M mutation in *ERG3*, while those with even higher MICs carried the L944P mutation in *RAD2* which is a DNA-damage repair-related gene. Differential expression analyses identified two upregulated (*IFF9* and *PGA6*) and three downregulated (*HGT7*, *HGT13*, and *PRI32*) genes in strains with reduced amphotericin B susceptibility (Tian et al. 2024).

Continued molecular epidemiology and functional validation research is essential for monitoring drug resistance trends and identifying novel mutations for the better understanding of multi-drug resistance in *C. auris* and the development of treatment strategies through coordinated efforts by public health agencies.

### 4.3. Aspergillus fumigatus

#### 4.3.1. Epidemiology and molecular characteristics

*Aspergillus fumigatus* is a filamentous ascomycete fungus and is widely distributed in the environment, such as the soil, decaying leaves, and composts. The conidia of *A. fumigatus* exist almost everywhere in the air and are being constantly inhaled by humans and other animals. The small size of the conidia, i.e. 2–3 μm in diameter, allows them to evade the mucociliary clearance in the respiratory system and reach the alveoli in the lung (Kwon-Chung and Sugui 2013). In healthy individuals, infections often do not establish due to the body’s immune defenses, while in immunocompromised patients, infections can occur in the lung area and may extend to other body parts (Kwon-Chung and Sugui 2013). It is estimated that *A. fumigatus* may be responsible for over 3 million chronic and invasive infections each year with a high mortality rate, ranging from 25% to 90% (Gsaller et al. 2016), representing a serious global health threat.

*A*. *fumigatus* has a haploid genome of around 29.4 million base pairs. Genetic analysis shows that this species exhibits extensive genetic diversity with both high level of recombination and clonality observed in different contexts (Ashu et al. 2017). The observed high genetic diversity likely results from a combination of local adaptation, sexual reproduction, and global dispersal (Ashu et al. 2017; Thorn and Xu 2023).

#### 4.3.2. Phenotypic features, diagnosis, and treatment

*A*. *fumigatus* grows as septate, colorless, and filamentous hyphae. It can reproduce both asexually and sexually, though sexual reproduction ability can vary widely among strains (O’Gorman et al. 2009; Korfanty et al. 2021). *A. fumigatus* prefers 37 °C but can grow at temperatures over 50 °C (Kwon-Chung and Sugui 2013). Remarkably, its ascospores are capable of surviving heat treatments up to 70 °C (Sugui et al. 2011). In common media, *A. fumigatus* forms white colonies which gradually turn blue-green or grey-green as conidia mature.

Aspergillosis is usually diagnosed with one or more of the following methods: imaging, galactomannan antigen detection, PCR, bronchoalveolar lavage, and biopsy (Segal and Walsh 2006; Cadena et al. 2021).

The most common treatment for aspergillosis is antifungal medication, with voriconazole being the first-line therapy (Segal and Walsh 2006; Fisher et al. 2022). For invasive aspergillosis, cocktail antifungal medication, such as voriconazole with echinocandins, may be used in situations involving antifungal resistance or pharmacokinetic variability (Panackal 2016; Fisher et al. 2022). For aspergillomas, surgery is usually suggested to remove the fungal mass to prevent bleeding (Piwkowski and Skrzypczak 2023).

#### 4.3.3. *Virulence factors in* A. fumigatus

*A*. *fumigatus* employs several key virulence factors to infect hosts, including those associated with and/or impacting adherence, thermotolerance, nutrition acquisition, cell wall, host-immune system interaction, and stress resistance (Earle et al. 2023).

Several adhesins have been identified playing a role in *A. fumigatus* host adherence which is a prerequisite for *A. fumigatus* infection. For example, RodA helps build the rodlet layer that makes the conidia surface hydrophobic and promotes binding to host collagen and albumin (Valsecchi et al. 2017). Other adhesins that enhance conidia/hyphae adherence involve the allergen Aspf2, the glycosylphosphatidylinositol anchored CspA protein, the extracellular thaumatin domain protein AfCalAp, and galactosaminogalactan GAG (Banerjee et al. 1998; Levdansky et al. 2007; Upadhyay et al. 2009; Speth et al. 2019).

*A. fumigatus* can adapt to a wide range of temperatures, facilitating its infection in humans. Two proteins are essential in *A. fumigatus* thermotolerance, α-1,2-mannosyltransferase and CgrA. Inactivation of either of these genes results in impaired growth at elevated temperatures and attenuated virulence in murine infection models (Bhabhra et al. 2004; Wagener et al. 2008).

*A. fumigatus* uses multiple nutrition acquisition strategies to survive sustainably in the hosts, either directly via enzyme-mediated tissue degradation, or indirectly through regulation of nutrient metabolism. Several functional genes have been identified as involved in these processes and related to virulence: the secreted serine protease gene *alp/aspf13*, which is involved in breaking down host tissues (Kolattukudy et al. 1993); *creA*, which regulates carbon utilisation (Beattie et al. 2017); *rhbA* and *cpcA* both linked to nitrogen regulation (Panepinto et al. 2003; Krappmann et al. 2004); *HapX*, which mediates adaptation to iron starvation by balancing iron uptake and usage (Schrettl and Haas 2011); and *zrfC* and *zrfA*, which control zinc uptake (Moreno et al. 2007; Amich et al. 2010).

Cell wall is a natural barrier for all fungi, shielding fungal cells from the external stresses. Not surprisingly, genes involved in *A. fumigatus* cell wall synthesis have been found to impact virulence as well. For example, deletions of the chitin synthase gene *chsG* (Mellado et al. 1996), the galactomannan synthase gene *glfA* (Schmalhorst et al. 2008), and the monosaccharide transporter gene *afmnt1* (Wagener et al. 2008) all caused the complete and/or partial reduction in *A. fumigatus* strain virulence.

*A*. *fumigatus* has various characteristics to enhance its defense against the host’s immune system. For example, *A. fumigatus* produces pigment to prevent cellular damage from reactive oxygen species. Deletion of the melanin biosynthesis gene *pksP* results in reduced virulence (Akoumianaki et al. 2016). Expression of anti-apoptotic protein AfBIR1 represses fungal programmed cell death *in vitro* (Shlezinger et al. 2017). Additionally, *A. fumigatus* can produce gliotoxin to weaken the host immune system. Deletion of gliotoxin-related genes such as *gliP* and *rglT* can affect *A. fumigatus* virulence (Kwon-Chung and Sugui 2008; Ries et al. 2020).

Sensing and responding to environmental changes are essential for the survival and reproduction of all organisms. In *A. fumigatus*, this process relies on the coordinated activity of various signaling pathways, such as the cAMP-PKA, cell wall integrity, high osmolarity glycerol, calcium-calcineurin, unfolded protein response, hypoxia response, and alkaline pH response pathways (Liebmann et al. 2004; Valiante et al. 2008; Dirr et al. 2010; Krishnan and Askew 2013; Askew 2014; Bertuzzi et al. 2014; de Castro et al. 2014; Juvvadi et al. 2014; Alves de Castro et al. 2016; Brown and Goldman 2016; Kowalski et al. 2019). Deletion or mutation in these genes has shown to impact *A. fumigatus* virulence.

Antifungal resistance has been extensively studied and reviewed for *A. fumigatus* (e.g., Fisher et al. 2022). Among the different categories of antifungal drugs, resistance to those in the azole category in *A. fumigatus* often involves genetic changes in *cyp51A* (Abdolrasouli et al. 2015), *SrbA* (van de Veerdonk et al. 2025), overexpression of ABC transporters (Rybak et al. 2018), etc.

#### 4.3.4. Research advances in China since 2022

Epidemiological research on *A. fumigatus* indicates a growing burden of *A. fumigatus-*associated aspergillosis in China, including antifungal resistance concerns. Studies have reported that invasive and chronic aspergillosis primarily occurs in elderly patients, those receiving immunosuppressant therapy, individuals in intensive care units, and patients with concomitant diseases. Commonly identified underlying comorbidities include pulmonary disease, gastroenteritis, haematologic malignancy, and cardiovascular disease (Wang et al. 2022a; Bilal et al. 2023a; Chen et al. 2024b). The overall frequency of antifungal resistance in clinical *A. fumigatus* strains remains low across the country, though variation exists between antifungal agents and geographic regions. Wang et al. (2022a) examined clinical *Aspergillus* strains collected between 2019 and 2021 in Anhui Province. They found the majority of the 156 isolates showed susceptibility to azoles, echinocandins, and amphotericin B, with MICs < 0.5 µg/mL, < 0.5 µg/mL, and 1.66–2.91 µg/mL, respectively. However, all *A. fumigatus* strains exhibit resistance to flucytosine (MICs > 64 µg/mL). In another study analyzing 276 *A. fumigatus* strains collected between 2021–2023 in Ningxia region, two azole-resistant strains were identified (Kang et al. 2024). Among 474 aspergillosis cases between 2017 and 2022 in South China, the wild-type *A. fumigatus* isolates against amphotericin B and triazoles were 99.1% and 97%–98% (Bilal et al. 2023a). Notably, *A. fumigatus* isolates from Yunnan presented a distinct resistance pattern. Among 94 clinical isolates, the resistance rates for itraconazole, voriconazole, and posaconazole were 16.0%, 21.3%, and 71.3% respectively, surpassing that observed in other parts of China. Meanwhile, a higher frequency of unique genotypes was observed among clinical Yunnan isolates than that from other parts of the country. Regarding the possible resistance origins, the authors pointed that certain clinical strains shared partial genotype similarities and resistance profiles with environmental strains (Gong et al. 2024a).

Different from the low frequency of drug resistance among clinical isolates, high frequencies of triazole resistance have been found among environmental isolates of *A. fumigatus* over the last five years. For example, about 80% of greenhouse isolates of *A. fumigatus* from near Kunming showed resistance to at least one triazole antifungal drug and many were cross-resistant to agricultural fungicides (Zhou et al. 2021). Lower frequencies but similar cross-resistance to multiple triazole antifungals have also been reported from agricultural fields, forests, and remote regions along the three parallel rivers in southwest China (Zhou et al. 2022a, 2023). Fundings from the current research progress in *A. fumigatus* epidemiology underscores the necessity of continued resistance surveillance and a deeper understanding of transmission dynamics, especially among populations at risk, to inform effective intervention strategies.

### 4.4. Candida albicans

#### 4.4.1. Epidemiology and molecular characteristics

*Candida albicans* (*C. albicans*) is a broadly distributed fungus, typically found on the skin and mucosal surfaces of humans and other mammals. However, this fungus can transition from a commensal to a pathogen under certain conditions, including the presence of underlying diseases, suppressed immune system, antibiotic usage, and the implantation of invasive medical devices (Lopes et al. 2015; Pendleton et al. 2017; Parambath et al. 2024). *C. albicans* can lead to a wide range of infections, from superficial to life-threatening systemic infections, and is one of the most prevalent yeast pathogens that cause invasive fungal infections (Parambath et al. 2024). Each year, *C. albicans* is estimated to cause over 150 million mucosal infections and roughly 200,000 deaths due to serious, body-wide infections in populations at high risks (Brown et al. 2012; Richardson 2022). According to a Canadian study, the 30-day mortality rate for *C. albicans* candidemia reaches 32.1% (95% confidence intervals: 25.9%–38.3%) (Bourassa-Blanchette et al. 2023).

*C*. *albicans* is a diploid, with a haploid genome size of approximately 14.5 million base pairs. It exhibits a high level of genomic diversity among strains. Analysis of 1,391 *C. albicans* isolates using multilocus sequence typing revealed 17 distinct clusters with each clade containing diverse sequence types, single-nucleotide polymorphisms, and signatures of recombination (Odds et al. 2007). The significant genetic diversity is attributed to multiple mechanisms, including loss of heterozygosity, ploidy shifts, chromosomal rearrangements, single nucleotide mutations, gene flow, and recombination (Bennett et al. 2014; Ropars et al. 2018; Avramovska and Hickman 2019; Ene et al. 2019; Mba et al. 2022). Such genetic plasticity provides *C. albicans* with enhanced ability to adapt to environmental stressors, evade host immune system, and develop antifungal resistance.

#### 4.4.2. Phenotypic features, diagnosis, and treatment

*C*. *albicans* can grow in different forms such as yeast, pseudo-hyphae, and hyphae. Some strains can switch from white to opaque and grey yeast colonies (Sudbery et al. 2004). The reproduction modes of *C. albicans* include asexual budding, heterothallic, and homothallic mating (Alby et al. 2009). However, meiosis and sexual spore production have not been reported in *C. albicans*. *C. albicans* can grow at temperatures between 20 °C and 40 °C, with yeast form growing optimally at 30 °C while hyphae growing best at 37 °C (Nadeem et al. 2013). On common fungal media such as Sabouraud dextrose agar, *C. albicans* typically forms smooth, white, creamy colonies.

Preliminary diagnosis of *C. albicans* can be accomplished using multiple methods, including microscopy, culture, antigen tests, and molecular tests. To distinguish among different *Candida* species, *C. albicans* germ tube antibody (Pietro et al. 2019) and molecular tests such as PCR and sequencing are needed.

Antifungal therapy is the main treatment option for *C. albicans* infections, and various routes of administration can be applied based on the site of infection and level of severity (CDC 2023; Cleveland Clinic 2025). For superficial, localized infections, pills, creams, suppositories, or tablets can be used, while systemic infections often require intravenous antifungals. Under conditions of severe infection, a combination of antifungal drugs may be applied.

#### 4.4.3. Virulence factors

Several virulence factors have been identified to impact *C. albicans* effectiveness in its pathogenesis: morphological transition, biofilm formation, and white-opaque switching. These are briefly described below.

Morphological transition of blastospores into hyphae allows *C. albicans* cells to enter the host tissue through penetration and induce endocytosis (Maza et al. 2017). The hyphae formation is mediated by several signal transduction pathways encompassing cAMP-dependent protein kinase A (Lin and Chen 2018). The hyphae cells have also been found to produce candidalysin which is capable of causing cytolysis of macrophages and dendritic cells (Kasper et al. 2018). Meanwhile, during hyphae formation, several adhesins such as Als3 and Hwp1 are expressed which augment the adhesion ability of *C. albicans* cells (Deorukhkar et al. 2014).

Biofilm is a virulence factor for the pathogenic success of *C. albicans*. The extracellular polysaccharide matrix plays a crucial role in biofilm formation and persistence and comprises several components. Notably, extracellular DNA allows *C. albicans* biofilm to bind and persist on abiotic surfaces, while β-1,3-glucans inhibit antibiotic penetration thereby increasing biofilm drug resistance (Karygianni et al. 2020; Talapko and Škrlec 2020). Several transcription factors (Bcr1, Efg1, Brg1, Ndt80, Rob1, and Tec1) have been identified as involved in regulating biofilm formation, development, and dispersion. *BCR1* and *EFG1* regulate several adhesin genes including *EAP1*, *HWP1*, and *ALS3*, which are essential in the formation of *C. albicans* biofilm (Ganguly and Mitchell 2011; McCall et al. 2019; Rodríguez-Cerdeira et al. 2020). *BRG1* binds to the 5’ regions of regulatory genes of hyphal development (Nobile et al. 2012). While *NDT80* is known as a transcriptional activator of biofilm formation and filamentous growth, it can also repress filamentous growth when the cells are under stress (Nobile et al. 2012). The above six transcriptional regulators form a dense interconnected transcriptional network in *C. albicans*, enabling coordinated control of biofilm formation and morphogenesis.

White-opaque switching has been linked to *C. albicans* virulence. The features of both cell types and their associations with virulence have been extensively studied (e.g. Anderson and Soll 1987; Bennett and Johnson 2005; Alby and Bennett 2009). Specifically, white cells are round, smooth, less adherent, and more virulent in systemic infections, while opaque cells are bean-shaped, flatter and more commonly associated with cutaneous infections. They also differ in mating ability and sensitivity to neutrophils, reactive oxygen species, and antifungal drugs. The white-opaque switching is controlled by a complex network of genes, among which the Wor family members are critical for initiating and maintaining the opaque cell state, especially *WOR1* (Zordan et al. 2006). Several transcription factors (e.g., Czf1, Cek1, and Sho1) promote the white-opaque switching process, while others (e.g. Msb2, Opy2, Ahr1, Efg1, and Ssn6) repress it (Sonneborn et al. 1999; Hernday et al. 2013, 2016; Brenes et al. 2020). In addition, the deletion of genes *NAT4*, *SET3*, or *HOS2* reduced the frequency of white-opaque switching, while *HST2* deletion increased reverse switching to the white state from opaque state (Hnisz et al. 2009). Furthermore, expression changes of other genes such as secreted aspartyl proteinases during the phenotypic switching were closely related to cell growth and proliferation under different environments (White et al. 1993).

#### 4.4.4. Research advances in China since 2022

In China, *C. albicans* has been the most commonly identified pathogen causing invasive fungal infections, consistent with the global pattern. Among invasive fungal infections, the proportions caused by *C. albicans* ranged from 11.7%–64.25%, depending on infection sites, study periods, and geographic areas (Bilal et al. 2022, 2023b; Hou et al. 2022; Song et al. 2022; Wen et al. 2022; Cai et al. 2023; Chen et al. 2023a, 2024a; Gao et al. 2023; Kan et al. 2023; Qiao et al. 2023; Chen and Weng 2024; Han et al. 2024; Li et al. 2024b; Liu et al. 2024; Wang et al. 2024a, 2024b; Zeng et al. 2024; Zhang et al. 2024b). Although this fungus remains the main cause of invasive fungal infections, there seemed a downward trend of *C. albicans* infection incidence. According to a two-phase surveillance by Yu et al. (2024), the proportion of *C. albicans* in intensive care units decreased significantly in Beijing during 2016–2017 compared to the period of 2012–2013. Moreover, according to a retrospective study spanning 2015–2023 by Li et al. (2024a), *C. tropicalis* (37.4%, 68/182) surpassed *C. albicans* (32.4%, 59/182) in causing candidemia in western China.

At present, the resistance rates of *C. albicans* to antifungal drugs remain generally low, particularly to the azole class. For example, among the 454 *C. albicans* isolates from the two-phase surveillance mentioned above by Yu et al. (2024), the resistant strains against fluconazole and voriconazole only accounted for 2.6% and 6.6% of the total strains, respectively. Notably, while the resistance rate did not rise in the second phase, the researchers observed that the MIC distribution metrics increased by two folds compared to those in the first phase. The gradual shift toward reduced susceptibility suggests the potential for a future rise in resistance, underscoring the need for continuing surveillance, appropriate antifungal stewardship, and cautious prescription practices.

Over the last few years, a major area of research in China on human fungal pathogens has been the search for natural products with antifungal, including anti-*Candida*, activities. Compounds from a wide range of organisms have been tested and found exhibiting antifungal bioactivity, including *Sarcococca hookeriana* var. *digyna* (common name: sweet box) (Shen et al. 2024), *Zanthoxylum simulans* (Chinese prickly ash) (Ma et al. 2022), *Macleaya cordata* (plume poppy) (Liu et al. 2023b), *Amycolatopsis* sp. YNNP 00208 (Qian et al. 2022), *Protaetia brevitarsis* (white-spotted flower chafer) (Fu et al. 2023), *Carex meyeriana* (sedge) (Du et al. 2024), and *Streptomyces microflavus* (Cao et al. 2024). The first four organisms even showed inhibition against fluconazole-resistant *C. albicans*. For example, molecular analyses revealed that the ethanol extracts of *Sarcococca hookeriana* var. *digyna* suppressed fluconazole-resistant *C. albicans* via sphingolipid metabolism and signaling pathway, while Chelerythrine from *Macleaya cordata* acted by decreasing cellular surface hydrophobicity and suppressing the cAMP pathway. With the increasing occurrence of drug-resistant *C. albicans* infections, identifying novel drug targets and the compounds with unique mechanisms of action is important for the discovery of effective alternatives to conventional therapies.

### 4.5. Other fungal pathogens

Beyond the critical fungal pathogens that dominate clinical research, several other fungi have also been frequently reported over the years from China, such as *Candida tropicalis*, *Talaromyces marneffei* (syn. *Penicillium marneffei*), *Mucor*, *Fusarium* spp., *Trichophyton indotineae*, etc. Although these pathogens are not considered critical or high priority pathogens, they pose significant health threats and challenges due to diagnostic difficulties, limited treatment options, and emerging resistance patterns in specific geographic regions, including certain regions in China. Below we describe a few high or medium priority fungal pathogens.

*Candida tropicalis* (*C. tropicalis*) is an opportunistic yeast pathogen of increasing importance. The pathogenicity of this fungus is primarily attributed to the ability to produce hyphae, form strong biofilm, and develop antifungal resistance (Marcos-Zambrano et al. 2014; Choi et al. 2016; Seneviratne et al. 2016; Zuza-Alves et al. 2017). It is the second frequently identified *Candida* species in clinical infections, particularly in tropical and subtropical areas and in immunocompromised individuals (Sharma and Chakrabarti 2023). In certain areas, such as India, *C. tropicalis* has surpassed *C. albicans* becoming the most frequently identified *Candida* species (Kaur et al. 2020). According to a systematic analysis across mainland China, *C. tropicalis* was the second most prevalent *Candida* species at ~21.9%, with the proportion varying across different regions (Bilal et al. 2022). Meanwhile, a steady increase in azole resistance in *C. tropicalis* has been reported in China (Fan et al. 2017). One recent study comparing the distribution of pathogens and drug resistance in bloodstream infections found that *C. tropicalis* was significantly increased from 19 (of 2,698 isolates) to 37 (of 2,922 isolates) during the COVID-19 pandemic than pre-pandemic (Gu et al. 2024).

*Talaromyces marneffei* (*T. marneffei*) is a thermally dimorphic fungus that can cause talaromycosis. This disease is endemic in Southeast Asia and southern China and frequently occurs in AIDS patients although sporadic cases in non-HIV patients have been reported (Vanittanakom et al. 2006; Chan et al. 2016; Castro-Lainez et al. 2018). The incidence of talaromycosis is increasing with the spread of HIV in Asia. In China, *T. marneffei* is mainly restricted in southern regions with more than 80% of cases reported in Guangxi and Guangdong provinces (Hu et al. 2013; Chen et al. 2017; Xie et al. 2022). Based on a retrospective cohort study on hospitalized AIDS patients in southern China, 16.1% of the 6,791 AIDS patients were co-infected by *T. marneffei* and the mortality was significantly higher in *T. marneffei*-infected group than *T. marneffei*-uninfected group, which was also the highest among all AIDS-associated complications (Jiang et al. 2019).

*Mucor* is a group of filamentous fungi that are commonly found in soil, decaying fruits and vegetables, and other organic materials. Some species are opportunistic human pathogens which can cause mucormycosis in various forms including cutaneous, rhino-orbital-cerebral, pulmonary, gastrointestinal, disseminated and other uncommon sites (Reid et al. 2020). Even for localized cutaneous diseases, mortality can reach 10%–30% (Reid et al. 2020). For invasive mucormycosis, mortality exceeds 30%–50%, and over 90% mortality is associated with disseminated disease (Reid et al. 2020). Populations at risk of mucormycosis are those with diabetes and compromised immune systems such as hematological malignancies patients, transplant recipients, and burn patients (Roden et al. 2005; Chakrabarti et al. 2006; Ledgard et al. 2008; Song et al. 2017). Prompt surgery and timely treatment with appropriate therapeutic agents are essential for improved outcomes. According to a case report in China, a male kidney recipient who experienced postoperative vascular rejection and Mucor-bacteria coinfection recovered after timely and appropriate treatment (Lian et al. 2024).

*Fusarium* (*F*.) is a genus of filamentous fungi that is abundant in the soil microbial community. Most species are harmless to healthy humans, but some can be pathogenic to plants and humans. For example, *F. solani*, *F. oxysporum*, *F. fujikuroi*, and *F. graminearum* can cause diseases in crops such as wilts and rots, representing a major concern for agriculture (Arie 2019; Xu 2022). Although not common, certain *Fusarium* species (e.g. *F. solani*, *F. oxysporum*, *F. verticillioides*, and *F. moniliforme*) can cause infections in humans, particularly in immunosuppressed individuals (Nucci and Anaissie 2007; Cighir et al. 2023). In immunocompetent individuals, *Fusarium* species can also cause superficial infections including keratitis and onychomycosis (Ferreira da Cunha Neto et al. 2024), though invasive and disseminated infections have also been reported, such as peritonitis in patients receiving dialysis, endophthalmitis osteomyelitis, fungemia, pneumonia, etc. (Sierra-Hoffman et al. 2005; Proença-Pina et al. 2010; Dananché et al. 2015; Poignon et al. 2020). In general, like many other fungal infections, severely immunocompromised patients are more likely to develop invasive or disseminated *Fusarium* infections (Nucci and Anaissie 2007; Cighir et al. 2023). Over the recent years, Chinese *Fusarium* research has been mainly revolved around its role as a plant pathogen, focusing on crop diseases and the development of control agents (Chen et al. 2023b; Dong et al. 2024; Gao et al. 2024; Khan et al. 2024; Son et al. 2024; Zhang et al. 2024a). Relatively few studies examined its impact on humans. According to a clinical epidemiological study investigating the distribution of infectious agents among severely burn-injured patients, 5.79% of 287 isolates were fungi and *Fusarium* (20.59%) was among the most commonly identified organisms, similar to those found for *Candida* (38.24%) and *Aspergillus fumigatus* (14.71%) (Jin et al. 2022).

*Trichophyton indotineae* (*T. indotineae*) is an emerging dermatophyte species that has recently gained global attention due to its association with widespread, recalcitrant dermatophytosis. *T. indotineae* infections often present with extensive skin lesions and a chronic relapsing course. Many *T. indotineae* strains were found resistant to terbinafine which is the first-line drug for treatment of moderate to severe dermatophytosis (Chowdhary et al. 2022). Originally reported in South Asia, particularly India, this fungus has been increasingly identified in other regions including Europe, Middle East, and North America, likely due to travel and migration (Fattahi et al. 2021; Chowdhary et al. 2022; Jabet et al. 2022; Ngo et al. 2022; Posso-De Los Rios et al. 2022). Although many infected patients have travel history to South Asia, *T. indotineae* infections were also reported in patients without known exposure history (Xie et al. 2024). Among the 14 multidrug-resistant *T. indotineae* cases in China, only one travelled abroad between June 2022 and August 2023 (Xie et al. 2024). Genomic analyses of Chinese isolated strains with strains from other countries suggested that *T. indotineae* was introduced into China multiple times and some strains experienced regional clonal transmission (Xie et al. 2025). The study also investigated the mechanism of *T. indotineae* resistance which is primarily linked to the Phe397Leu substitution in *SQLE*, whereas azole resistance is associated with *CYP51B* copy number increase together with upregulated *MDR2* and *MDR3* expression.

Even though these fungal pathogens are not currently classified as critical, they have the potential to become so if their prevalence increases, infections become difficult to treat, and/or they acquire additional virulence or resistance traits. Therefore, ongoing research and surveillance, including studies of risk populations, virulence factors, novel drug targets and drugs, and antifungal resistance patterns are needed to monitor, understand, and control these important pathogens.

## 5. Prevention and control strategies of fungal infections

The rapid advancement of medical technologies, including organ transplantation, broad-spectrum antibiotics, and immunosuppressive agents, has significantly altered patient demographics. Coupled with population aging, this has led to a steadily increasing number of immunocompromised individuals in China (e.g. Lee and Lau 2017; Liu et al. 2021; Sun et al. 2024). Consequently, the incidence and mortality rates of invasive fungal diseases (IFDs) have risen significantly, with these infections now representing a leading cause of direct mortality among patients with malignancies, organ transplants, and other underlying conditions. Meanwhile, common fungal diseases, such as dermatophytosis and candidal vulvovaginitis, are also highly prevalent, substantially impacting patients’ quality of life and contributing to a considerable public health burden (Xu 2022; Bhosale et al. 2025; Li et al. 2025a). Compared to post-diagnosis treatment, prevention and control strategies play an increasingly critical role in reducing infection risks. These strategies can markedly reduce the risk of infection among high-risk populations, alleviate patient suffering and healthcare expenditures, prevent the need for complex interventions, and significantly contribute to improved clinical outcomes and more efficient use of healthcare resources.

### 5.1. Public health measures

One issue that requires urgent attention is the longstanding inadequacy of public health measures for fungal diseases on a global scale. This insufficiency is largely attributable to the intrinsic characteristics of fungal infections—for example, many severe fungal infections (such as IFDs) primarily affect individuals with compromised immune systems, rather than those with normal immune function. However, in recent years, there have been positive developments in this area. In 2022, WHO released the WHO FPPL to guide research, development, and public health action, which conducted a public health risk assessment of pathogenic fungi at a global level for the first time. The WHO FPPL has garnered widespread attention in China, sparking in-depth discussions (e.g. Tang et al. 2024). In principle, public health measures for fungal diseases are also applicable under the Infectious Disease Prevention and Control Law of the People’s Republic of China (NHC 2023; China SPP 2025). This law adopts a policy of “prevention first, combining prevention with treatment,” and adheres to the principles of law-based and science-based disease control strategies. By codifying these measures, the law ensures the rapid and uniform implementation of public health interventions nationwide.

In China, public health issues related to pathogenic fungi mainly involve endemic fungal infections and antifungal drug resistance (Gong et al. 2023). Endemic fungal diseases in China primarily include histoplasmosis, talaromycosis, and sporotrichosis. Antifungal drug resistance, on the other hand, mainly concerns more common fungal pathogens such as *Aspergillus* spp., *Candida* spp., and dermatophytes. The prevention and control of endemic fungal infections have mainly focused on high-risk populations or regions with high incidence rates. More specifically, histoplasmosis and talaromycosis are primarily found in southern China, while sporotrichosis is more common in northeastern China. Therefore, different diseases require different key areas for prevention and control. In comparison, antifungal resistance poses a nationwide challenge. Since 2022, both antifungal resistance and antibacterial resistance have been guided by the government document “National Action Plan to Contain Antimicrobial Resistance (2022–2025)”. In line with the principles outlined in this document, different regions and sectors have formulated their own work plans accordingly. For example, both Yunnan Province and Fujian Province have issued their own localized “Action Plans to Contain Antimicrobial Resistance (2023–2025)”.

It is particularly noteworthy that the publication of the Consensus for Antifungal Stewardship in China (2024 Edition) provides an authoritative guidance framework and implementation pathway for systematically addressing the increasingly severe issue of antifungal drug resistance (Ye et al. 2024). By advocating for standardized and individualized antifungal drug use strategies, this consensus has significantly reduced the rates of empirical, excessive, and inappropriate use of antifungal agents. As a result, it has directly alleviated the selective pressure on fungal pathogens, potentially delaying the emergence and spread of drug-resistant strains. The implementation of this consensus will help optimize the clinical use of antifungal agents, prolong the clinical lifespan of existing antifungal drugs, and buy valuable time for the development of new antifungal agents. Ultimately, it should have a profound impact on ensuring patient safety, reducing treatment failure rates associated with drug resistance, and alleviating the public health burden.

### 5.2. Vaccination and immunoprophylaxis

Fungal infections have emerged as a significant public health threat; however, current antifungal therapies remain inadequate for effective management. One reason lies in the inherent toxicity and high cost of some of these last-line drugs; another is the emergence of varying degrees of resistance to all currently available antifungal agents. In addition to developing new antifungal drugs, there is great anticipation for the development of fungal vaccines, which could substantially improve the health outcomes of populations at high risk for fungal infections. However, there are currently no licensed fungal vaccines available. As no licensed fungal vaccines are currently available, immunoprophylactic approaches for fungal diseases have yet to be implemented (Rivera et al. 2022; Gong et al. 2024b).

The intended use scenarios of fungal vaccines mean that their development will face certain technical challenges not typically encountered in the development of traditional vaccines for bacterial and viral infections. Most severe fungal infections occur in immunocompromised individuals. This indicates that fungal vaccines must be effective in hosts with compromised immune function. Moreover, it is of particular importance that immunocompromised individuals face a clinical risk of fungal infections that may originate from multiple different pathogenic fungi. This suggests that an ideal fungal vaccine should preferably provide protection to the recipient against multiple invasive fungal pathogens. Finally, pathogenic fungi, as complex eukaryotic organisms with intricate cell wall structures, also present increased difficulty in identifying antigenic factors for vaccine development.

Although the development of fungal vaccines is challenging, the expected market size for such vaccines is relatively small compared to those for viruses and bacteria. In most cases, only immunocompromised individuals require fungal vaccination. The smaller anticipated market size also reduces the willingness of vaccine developers and manufacturers to invest in this area.

In the case of China, the demand for vaccines against *Candida* spp., *Aspergillus* spp., *Cryptococcus* spp., and *Talaromyces marneffei* may be the highest. According to published studies, research on vaccines for *Candida* spp., *Aspergillus* spp., and *Cryptococcus* spp. has been increasing, which may be due to the global distribution of these three pathogenic fungi (Sui et al. 2017; Del Bino and Romano 2020; Ivanov et al. 2022; Sun et al. 2023; Zhai et al. 2024). In contrast, vaccine research for *Talaromyces marneffei* remains relatively limited, possibly because its geographical distribution is more restricted to Southeast Asia and southern China. Therefore, vaccine development targeting *Talaromyces marneffei* should receive greater attention from Chinese researchers and vaccine manufacturers.

### 5.3. Environmental control and sanitation

In most cases, pathogenic fungi are transmitted through an “environment-to-human” route of infection. Therefore, environmental control and sanitation play a more significant role in the prevention and control of pathogenic fungal infections, compared to bacterial and viral pathogens. In China, current efforts in this domain are primarily manifested through hygiene inspections in public places—particularly those frequented by immunocompromised individuals, such as hospital wards and nursing homes.

Specifically, China has established detailed standards and management regulations for monitoring the hygiene conditions of public places, such as the Regulations on Hygiene Management in Public Places and its implementing rules (NHC 2011; MIIT 2020). For special areas like hospital wards, more stringent hospital infection control measures have been put in place. These measures may differ slightly across hospitals, primarily due to regional variations in the risk of fungal infections within China. As a result, many general protocols may require further optimization when implemented in order to suit specific local conditions.

Among the many concerns on control measures, the most pressing one relates to optimization of validation protocols of existing disinfection guidelines, particularly on the specific challenges posed by pathogenic fungi. Due to factors such as the fungal cell wall, the survival capacity of pathogenic fungi in the environment is generally higher than that of bacteria and viruses. This implies that the disinfection techniques and protocols required for pathogenic fungi need to be specifically validated. Currently, the main fungal species used for validation of disinfection methods are *C. albicans* and *A. niger*. These two species represent morphologically distinct types: yeast-like fungi and filamentous fungi, respectively. However, considering the high diversity of pathogenic fungi (approximately 500 species are currently known) and the significant differences in their taxonomy and phenotypes (NHC 2023; Zhao et al. 2024), it may be necessary to include a broader range of fungal species for the validation of disinfection technologies.

In addition to specific environmental control measures, efforts in the prevention and control of pathogenic fungi should also pay attention to the issue of antifungal resistance in the environment. The widespread use of antifungal agents in industries such as agriculture and animal husbandry has led to a rapid increase in drug-resistant fungi in the environment (MIIT 2010; NHC 2020). These drug-resistant fungi may eventually become serious public health concerns. Addressing this issue requires the joint efforts of experts from multiple disciplines.

### 5.4. Surveillance and early warning systems

Currently in China, specialised fungal disease surveillance initiatives mainly include the following networks: the National Monitoring Network for Fungal Diseases, the China Surveillance Network for Dermatophytes Resistant to Antifungal Agents, and the National Surveillance Network for Cutaneous Fungal Diseases. In addition, fungal pathogen surveillance has also been incorporated into the national sentinel surveillance system for acute respiratory infectious diseases, which is led by China CDC. This system specifically covers respiratory infections caused by *Aspergillus* spp. and *Cryptococcus* spp. These monitoring systems each have their own focus areas and have made substantial progress within their respective fields (NHC 2019; FAHGMU 2025). For instance, the national monitoring network for fungal diseases has observed, through long-term surveillance of antifungal resistance among pathogenic fungi, a sharp rise in azole resistance rates among *C. tropicalis* in recent years (Brueggemann et al. 2021; Bing et al. 2024a; Huang et al. 2024). Moreover, it has been demonstrated that this increase in azole resistance is closely correlated with the rapid expansion of the AZR cluster. In addition, scientific research-oriented monitoring is also in place, but these efforts mainly focus on specific pathogenic fungi, such as *C. auris*, *Talaromyces marneffei*, and *Sporothrix globosa* (Du et al. 2022; Wang et al. 2022b; Chen et al. 2023a; Bing et al. 2024b; Deng et al. 2024).

Among these surveillance efforts, the greatest challenge lies in the standardization and normalization of monitoring technologies. Due to the uneven technical capabilities across different surveillance sites, the reliability of the monitoring data is somewhat compromised and difficult to compare. Furthermore, the inherent complexity of pathogenic fungi adds another layer of difficulty to surveillance. The chitin-rich fungal cell walls hinder DNA extraction, complicating molecular detection techniques such as PCR. At the same time, fungal spores also increase the risk of laboratory contamination, which is particularly evident in the surveillance of filamentous fungi. Because fungi are eukaryotic organisms with relatively complex life processes, it is difficult to directly predict phenotypic traits—such as drug resistance—based solely on genetic data. Therefore, surveillance sites typically need to isolate strains through cultivation before conducting in-depth analysis. All of these factors make fungal surveillance work challenging.

To effectively address the potential issues currently existing in pathogenic fungal surveillance, an integrated systematic approach is needed. This involves not only seeking purely technical solutions, but also establishing a multi-agency collaboration mechanism, strengthening the development of specialized teams, integrating and optimising resources, and continuously adjusting and refining strategies through long-term practice.

## 6. Antifungal stress response

Antifungal failure may arise from different mechanisms, including resistance, tolerance, and persistence (Delarze and Sanglard 2015). Resistance occurs when fungi survive and reproduce during antifungal treatment, which falls into two categories: intrinsic and acquired resistance. Intrinsic resistance refers to the natural insensitivity of a species to a drug without prior exposure or genetic mutation, while acquired resistance is defined as a new resistance trait gained by a previously drug-susceptible species or strain after exposure to the drug, usually via mutation. For instance, *C. krusei* has an intrinsic low susceptibility to fluconazole due to a reduced sensitivity of the target enzyme cytochrome P450 sterol 14 alpha-demethylase to inhibition by this drug (Orozco et al. 1998). *C. glabrata* is intrinsically resistant to fluconazole, with many clinical isolates exhibiting a 16–64 folds higher fluconazole MIC than *C. albicans*, mainly due to the overexpression of multidrug resistance transporters (e.g., *CDR1*, *CDR2*, and *SNQ2*) regulated by the transcription factor gene *PDR1* (Schulz et al. 2012).

In contrast, acquired resistance is commonly developed through genetic changes, followed by selection, particularly long-term drug selection either in natural environment or within hosts. In *C. albicans*, azole resistance can be acquired through mutations in the drug target gene *ERG11*, overexpression of drug efflux pumps such as *MDR1*, and mutations in transcription factors like *UPC2* (Costa-de-oliveira and Rodrigues 2020).

Compared to resistance, tolerance, and persistence are overlooked aspects in terms of the efficacy of a given drug. Drug tolerance refers to the ability of a species to survive the drug concentrations above the MIC (Fridman et al. 2014; Delarze and Sanglard 2015). Drug tolerance is due to transient and reversible mechanisms and may not be stable, different from drug resistance which is the result of stable genetic changes. Antifungal tolerance is widespread. As noted by Delarze and Sanglard (2015), fungal pathogens are generally tolerant to azoles as many fungal cells can withstand drug concentrations above their MICs. Antifungal tolerance can be induced by multiple mechanisms including stress pathway responses (Berman and Krysan 2020), epigenetic alterations (Rosenberg et al. 2018), biofilm protection (Kean and Ramage 2019), and aneuploidy (Yang et al. 2021). Notably, antifungal tolerance can be reversed, typically after removal of the drug pressure.

Persistence is another phenotypic adaptation that usually emerged from drug tolerance. The cells that survive at a drug concentration higher than the MIC are defined as persisters. Persisters enter a dormant or quiescent state during antifungal treatment, but they are not mutated and can resume growth once the drug is removed (Wuyts et al. 2018). Therefore, persisters often underlie chronic and recurrent infections, requiring novel treatment options. Identifying key biomarkers and untangling resistance, tolerance, and persistence is crucial for understanding fungal drug responses and improving therapeutic outcomes for patients (Thorn and Xu 2025).

## 7. Antifungal pipeline: from historical stagnation to a new era of innovation

### 7.1. Brief historical perspective

The history of antifungal drug discovery is marked by an enduring slowness when compared to other areas of antimicrobial development. For example, the first antibacterial agent, penicillin, was introduced for clinical use in 1941, while the first antifungal agent, Nystatin, was not discovered until 1950 (Sousa et al. 2023). The antifungal armamentarium that exists today is largely a product of this slow historical development, with only five distinct drug classes dominating clinical use: polyenes, pyrimidine analogs, azoles, allylamines, and echinocandins (Vanreppelen et al. 2023). The most recent major addition, the echinocandin class, was introduced over two decades ago (Vanreppelen et al. 2023). This reliance on a small number of drug classes for the past fifty years has been the primary driver of the narrative of stagnation, particularly in the face of rapidly evolving fungal resistance (Schinas et al. 2024).

The enduring stagnation in antifungal pipeline is not due to a lack of effort but is a direct consequence of a fundamental biological and economic challenge. Unlike bacteria, which have distinct cellular targets, fungi are eukaryotes, sharing many cellular and metabolic pathways with humans (Roemer and Krysan 2014). This inherent similarity makes it exceptionally difficult to identify a fungicidal compound that will not also cause significant host toxicity. The high degree of biological overlap between pathogen and host creates a significant barrier to entry for new, safe compounds, making the development process longer, more expensive, and more prone to failure. These biological challenges are compounded by significant economic barriers. The research and development of a new antifungal drug is a high-cost, high-risk endeavor with uncertain market returns, particularly when compared to other therapeutic areas such as antiviral, antibacterial, and non-infectious diseases (Schinas et al. 2024). The combination of a high-risk development profile and uncertain market returns has historically disincentivised private sector investment, contributing directly to the “dry spell” in the pipeline.

### 7.2. Recent global developments

Luckily, the current antifungal pipeline is no longer dominated by incremental improvements to existing drug classes, such as Rezafungin, a next-generation echinocandin with an exceptionally long half-life of approximately 133 h (The Lancet Infectious 2023; Andes et al. 2025), and Isavuconazonium sulfate (Cresemba). Isavuconazonium sulfate is an advanced azole antifungal agent that was approved by the FDA for treating invasive aspergillosis and mucormycosis. It employs an entirely novel mechanism of action, offering a path forward in the face of widespread resistance against current azoles. Furthermore, a recent WHO pipeline review identified a total of 43 antifungal products in clinical and preclinical development as of September 2024. Of the 21 agents in clinical development, 9 are considered novel (WHO 2025). It features several first-in-class agents in development, such as Olorofim (an orotomide) and Fosmanogepix (an oxazole), which employ novel mechanisms to target WHO priority pathogens, including azole-resistant molds. Simultaneously, strategies to enhance existing therapies are being pursued, including the development of inhaled formulations like Opelconazole to improve lung targeting and the investigation of label extensions for approved drugs to treat more severe invasive infections. Detailed information on specific agents is provided below.

Ibrexafungerp (Triterpenoids): Ibrexafungerp is a first-in-class antifungal and the first representative of the novel triterpenoid class (Team 2025). It operates as a glucan synthase inhibitor, a mechanism similar to echinocandins, but with the distinct advantage of being available in an oral formulation (GSK 2023). This provides a new, flexible option for patients who require oral antifungal treatment. The US Food and Drug Administration (FDA) approved ibrexafungerp for the treatment of vulvovaginal candidiasis and the reduction of recurrent infections (GSK 2023). Its broad-spectrum activity against multidrug-resistant pathogens, including those resistant to both azoles and echinocandins, has also led to its late-stage investigation for life-threatening invasive fungal infections.

Olorofim (Ortomides): Representing a completely new class of antifungals called ortomides, olorofim inhibits the fungal dihydroorotate dehydrogenase (DHODH) enzyme, which is essential for *de novo* pyrimidine biosynthesis. The selective inhibition of fungal DHODH, with limited effect on human DHODH, disrupts the production of pyrimidines, which are vital for DNA and RNA synthesis, and consequently arrests fungal pathogen growth (The Lancet Infectious 2023; Puumala et al. 2024). This novel mechanism of action offers a much-needed new target, distinct from the cell wall and membrane pathways of traditional therapies. Olorofim has demonstrated activity against several clinically important groups of fungi, including *Histoplasma* spp. and *Aspergillus* spp. (The Lancet Infectious 2023; Schinas et al. 2024).

Fosmanogepix: Fosmanogepix is another first-in-class, broad-spectrum antifungal that is now being tested in a Phase 3 trial (Schinas et al. 2024; Zobi and Algul 2025). The drug targets and inhibits an enzyme in the glycosylphosphatidylinositol (GPI) anchor biosynthesis pathway, a mechanism that is both novel and distinct from all existing antifungal therapies. This novel mode of action is particularly promising as it has shown efficacy against a broad range of fungal pathogens, including difficult-to-treat, multidrug-resistant strains like *Candida auris* and *Aspergillus* species (The Lancet Infectious 2023).

DT-23: DT-23 is an inositol polyphosphate kinase (IPK) inhibitor that represents a new class of antifungal drugs with a novel mechanism of action (Desmarini et al. 2025). Researchers have shown that DT-23 has antifungal activity against the meningitis-causing fungal pathogen *Cryptococcus neoformans* and, more importantly, that it has a dual-targeting action that could prevent fungi from becoming resistant to the drug. This dual-targeting strategy has the potential to overcome a key limitation of existing drug classes. Other promising candidates include ME1111, a succinate dehydrogenase inhibitor being developed for onychomycosis, and a range of non-traditional agents in preclinical stages, including six that fall outside of conventional classes (WHO 2025). Table 1 shows some key new antifungal agents in development.

**Table 1.** Recent developments in antifungal agents.

Drug name	Class	Mechanism of action	Clinical/regulatory status
Ibrexafungerp	Triterpenoid	Inhibits glucan synthase	FDA-approved for VVC; Phase 3 for IC/IA
Olorofim	Ortomide	Inhibits fungal pyrimidine synthesis	Phase 2b; QIDP/Breakthrough designation
Fosmanogepix	Novel	Inhibits GPI anchor biosynthesis	Phase 3
Rezafungin	Echinocandin	Inhibits β-1,3-D-glucan synthase	FDA-approved for candidemia/IC
DT-23	Novel	Inhibits inositol polyphosphate kinases	Preclinical
ME1111	Novel	Inhibits succinate dehydrogenase	Clinical development

### 7.3. Developments in China

The global effort to combat fungal pathogens and replenish the drug pipeline is not limited to Western pharmaceutical companies. China, in particular, is emerging as a significant, and often overlooked, contributor through both its long-standing tradition of herbal medicine and its rapidly maturing modern pharmaceutical industry.

Traditional Chinese Medicine (TCM) represents a vast and largely untapped library of natural products with documented antifungal effects. This empirical legacy provides a unique and parallel approach to drug discovery that complements modern synthetic chemistry. The formula known as Cao Huang Gui Xiang (CHGX), for example, is a Chinese herbal medicine that has been used for more than two decades in the clinical treatment of severe *Candida* infections (Yue et al. 2022). Recent studies have validated its anti-*Candida* properties *in vitro* and have demonstrated its *in vivo* efficacy in mice with no noticeable toxicity at clinical concentrations (Yue et al. 2022). The mechanisms of action of CHGX are multi-pronged, including the disruption of cell membrane integrity, inhibition of biofilm formation and filament development, and the triggering of reactive oxygen species (ROS) accumulation that leads to rapid fungal cell death (Yue et al. 2022). The complex composition of CHGX and its ability to act on multiple pathways simultaneously may provide an effective strategy for overcoming resistance.

Another notable example of antifungal agents in China is pseudolaric acid B, a bioactive compound extracted from the root bark of the Chinese plant *Pseudolarix kaempferi*. This compound has been used as an antifungal remedy in TCM for thousands of years (Yan et al. 2012). Modern *in vitro* studies have shown that it possesses potent anticandidal activity, similar to fluconazole, against various *Candida* species, including some non-albicans strains that are inherently resistant to first-line antifungals (Yan et al. 2012). Researchers have also observed a synergistic effect when pseudolaric acid B is combined with fluconazole, highlighting its potential for use in combination therapy (Yan et al. 2012).

Beyond these, hundreds of other TCMs and their isolated compounds have shown antifungal effects with potential novel mechanisms of actions. For instance, the terpenoid eucarobustol E, isolated from *Eucalyptus robusta*, inhibits fungal growth by negatively regulating carbon flow towards ergosterol, a mechanism that is fundamentally different from existing antifungal drugs (Zhong et al. 2023). This rich empirical history and the ongoing research to validate and understand these natural products position TCM as a valuable source of inspiration for future drug discovery.

The expanded search for new antifungals in TCM and other sources is being facilitated by artificial intelligence (AI), with promise to fundamentally transform the drug discovery process. AI algorithms can analyze vast datasets to identify novel compounds, optimize drug design, and predict resistance mechanisms at a speed and scale impossible with traditional methods (Li et al. 2025b). A study led by researchers from Shandong University in China demonstrated this potential by using a two-step AI model to identify new protein compounds, including one with potent activity against the multidrug-resistant fungus *Nakaseomyces glabratus* (Li et al. 2025b). This success demonstrates how AI can overcome the limitations of traditional screening and accelerate the identification of promising drug candidates, offering a new, more efficient paradigm for combating antimicrobial resistance.

Despite the vibrant scientific progress in the antifungal drug pipeline, the reality of clinical availability remains limited. The translation of a promising laboratory compound into a widely accessible treatment remains a systemic challenge rooted in regulatory hurdles and economic realities.

## 8. Conclusions and perspectives

In this paper, we analyzed the temporal patterns of medical mycology research in China based on bibliographic data in two major scientific literature databases, the WOSCC database representing research published in English language from 1983 to 2024 and CNKI representing those published in Chinese language from 1991–2024. Our analyses showed increasing contributions in the WOSCC database by university-based Chinese researchers from 1983 to 2024 and in the CNKI database from hospital-based researchers that peaked in 2007 but has remained relatively steady since then. Linkages among keywords, researchers, and institutions within individual databases revealed enhanced interdisciplinary research activities and increasing collaborations with international colleagues over time. In addition, we briefly described the four critical priority fungal pathogens *C. neoformans*, *C. auris*, *C. albicans*, and *A. fumigatus*, and highlighted contributions by Chinese researchers towards understanding the four pathogens since 2022 when WHO declared them as critical priority. A shared challenge for treating all fungal infections is the emergence and spread of antifungal resistance. We briefly described antifungal resistance, tolerance, and persistence, and provided an update on recent developments in antifungal drug pipelines, including those by Chinese researchers. As recommended by the WHO on how to meet the challenges posed by fungal pathogens, China has made significant progresses in public health measures against fungal pathogens through targeted surveillance, fast and accurate diagnosis, developing new antifungals, and sound antifungal stewardship in both clinics and agriculture. However, significant gaps remain, including developing effective guidelines for reducing hospital and community acquired fungal infections, identifying regional specificity in disease agents and transmissions, and ultimately developing targeted warning and personalized treatments.

## Supplementary Material

Supplemental Material
